# Advancing DNA Barcoding to Elucidate Elasmobranch Biodiversity in Malaysian Waters

**DOI:** 10.3390/ani13061002

**Published:** 2023-03-09

**Authors:** Kar-Hoe Loh, Kean-Chong Lim, Amy Yee-Hui Then, Serena Adam, Amanda Jhu-Xhin Leung, Wenjia Hu, Chui Wei Bong, Aijun Wang, Ahemad Sade, Jamil Musel, Jianguo Du

**Affiliations:** 1 Institute of Ocean and Earth Sciences, Universiti Malaya, Kuala Lumpur 50603, Malaysia; 2 Institute of Biological Sciences, Faculty of Science, Universiti Malaya, Kuala Lumpur 50603, Malaysia; 3 World Wide Fund for Nature Malaysia, Petaling Jaya 46150, Malaysia; 4 Key Laboratory of Marine Ecological Conservation and Restoration, Third Institute of Oceanography, Ministry of Natural Resources, Xiamen 361005, China; 5 Fujian Provincial Key Laboratory of Marine Physical and Geological Processes, Xiamen 361005, China; 6 Laboratory of Coastal and Marine Geology, Third Institute of Oceanography, Ministry of Natural Resources, Xiamen 361005, China; 7 Department of Fisheries Sabah, Kota Kinabalu 88624, Malaysia; 8 Fisheries Research Institute Sarawak, Department of Fisheries Malaysia, Kuching 93744, Malaysia; 9 Xiamen Ocean Vocational College, Xiamen 361100, China

**Keywords:** cytochrome c oxidase 1, species identification, DNA barcode, reference library, phylogenetic tree, shark and ray

## Abstract

**Simple Summary:**

One-third of shark and ray species are threatened due to overfishing, but a lack of information on each species makes conservation decisions difficult. To address this issue, we conducted a study to identify the different species of sharks and rays in Malaysian waters using DNA barcoding of the CO1 gene, which is akin to DNA fingerprinting for species. We collected 175 individuals between June 2015 and June 2022, randomly selecting up to six specimens from each species. We successfully generated DNA barcodes for 67 species, belonging to 44 genera, 20 families, and 11 orders. Accurate species identification will improve species-specific catch landing data and accelerate the identification of use and illegal trade in Malaysia.

**Abstract:**

The data provided in this article are partial fragments of the Cytochrome c oxidase subunit 1 mitochondrial gene (CO1) sequences of 175 tissues sampled from sharks and batoids collected from Malaysian waters, from June 2015 to June 2022. The barcoding was done randomly for six specimens from each species, so as to authenticate the code. We generated barcodes for 67 different species in 20 families and 11 orders. DNA was extracted from the tissue samples following the Chelex protocols and amplified by polymerase chain reaction (PCR) using the barcoding universal primers FishF2 and FishR2. A total of 654 base pairs (bp) of barcode CO1 gene from 175 samples were sequenced and analysed. The genetic sequences were blasted into the NCBI GenBank and Barcode of Life Data System (BOLD). A review of the blast search confirmed that there were 68 valid species of sharks and batoids that occurred in Malaysian waters. We provided the data of the COI gene mid-point rooting phylogenetic relation trees and analysed the genetic distances among infra-class and order, intra-species, inter-specific, inter-genus, inter-familiar, and inter-order. We confirmed the addition of *Squalus edmundsi*, *Carcharhinus amboinensis*, *Alopias superciliosus,* and *Myliobatis hamlyni* as new records for Malaysia. The establishment of a comprehensive CO1 database for sharks and batoids will help facilitate the rapid monitoring and assessment of elasmobranch fisheries using environmental DNA methods.

## 1. Introduction

Elasmobranchs, a subclass of Chondrichthyans that include sharks and batoids (rays, skates, guitarfish, and sawfish), are widely distributed in the Southeast Asian (SEA) region and encompass more than 300 species from freshwater environments to deep seas [1]. Within the SEA region, Malaysia is one of the top countries with rich biodiversity of elasmobranch species. To date, there are at least 70 species (19 families) of sharks and 91 species (11 families) of batoids that have been reported to occur in Malaysian waters (Supplementary Materials Table S1) [2,3,4,5,6,7,8,9,10,11,12,13,14,15,16,17]. Although new species are continuously discovered, the populations of sharks and batoids have gradually decreased over the past decades [18]. The reduction in the population sizes of sharks and batoids is mainly due to high bycatch rates within commercial fisheries and recreational fishing activities [19]. Additionally, due to their K-selected life histories, some elasmobranch species are threatened with extinction [20,21]. Malaysia is the world’s ninth largest producer of shark products, especially shark fins, and the second largest importer in terms of volume, as reported by the Food and Agriculture Organisation (FAO) of the United Nations [22]. It is also one of the major countries globally with high annual elasmobranch landings [8]. These sharks and batoids are generally landed whole and are mostly fully utilized. According to the reported annual fish landings in Malaysia (http://www.dof.gov.my, accessed on 5 January 2023), the biomass of sharks and batoids reduced around 15-fold (6487 tonnes to 438 tonnes) and 9-fold (11,993 tonnes to 1372 tonnes), respectively, from 2018 to 2021 [18,23,24]. These data suggested the depletion of shark and batoid populations in Malaysian waters, parallel to the findings on global shark and batoid landings [20,25]. However, as explained by the Department of Fisheries Malaysia (DOFM) [26], sharks are not specifically targeted by fishers in Malaysia, but they are caught as bycatch along with other commercially important species by various fishing gears.

Globally, the estimated species numbers of elasmobranchs threatened with extinction have increased from approximately one-quarter [20] to more than one-third [27] due to targeted and incidental overfishing. This can also be explained by their conservative life history traits, including slow growth, long lifespan, late maturity, and low fecundity, that render elasmobranch populations difficult to recover from anthropogenic pressures [20,21,28,29]. The decrease in elasmobranch populations demonstrated the urgent need for conservation and management plans [20,21,25,30,31]. Due to the lack of species-specific information and variability of population sizes among species, the current landing records fail to provide details as to which species require protection the most. Accurate species identification is one of the most important elements to take into consideration in order to carry out suitable conservation and management programmes. The field identification of several closely related sharks (including carcharhinid, sphyrnid, and triakid sharks) and batoids (Myliobatiformes and skates) is often challenging, which might result in inaccurate species compositions and diversity in catch reports [32,33]. Moreover, only a few detailed studies have been conducted on the taxonomy and diversity of elasmobranchs in Malaysia [9,34,35]. The lack of data in this field is mainly due to large specimen sizes, ethical reasons, lack of experienced taxonomists, and high field survey costs, which, in turn, render accurate identification more challenging [36]. Thus, there is the potential that many undiscovered species remain unknown. A simple and accurate species identification tool is therefore crucial to allow the species-specific development of fishery conservation and management plans, especially those pertaining to the mislabelling and misidentification of shark and batoid products [12].

Over the past two decades, DNA barcoding has been introduced for its efficiency and accuracy in the identification of challenging species from different taxa [37,38]. The idea of this approach is conceptually straightforward—it uses a short DNA fragment of a specific gene of an approximately 650 bp region to be compared against reference sequences for accurate animal species identification [38]. The Cytochrome c oxidase subunit 1 mitochondrial gene (CO1) is suggested as a highly suitable marker for DNA barcoding, as it can discriminate between closely related species across diverse animal phyla [36], including sharks and rays [39,40]. To date, DNA barcoding has been successful in identifying elasmobranch species from dried fins, tissues, and carcasses [41,42,43,44,45,46,47]. This is highly beneficial for non-taxonomists to identify species with reasonable confidence even in the absence of whole specimens. Species can be identified by comparing their DNA barcode sequences against an online repository of barcode references, such as the NCBI GenBank (https://www.ncbi.nlm.nih.gov/genbank, accessed on 5 January 2023) and Barcode of Life Data System (BOLD) (http://www.boldsystems.org, accessed on 5 January 2023) [38,48,49]. However, DNA barcoding has limitations, such as the possibility of errors due to poor DNA quality or contamination, the potential for hybridisation or incomplete lineage sorting, and the lack of universality of CO1 primers across different taxonomic groups.

DNA barcoding—more specifically, CO1 analysis—has been used widely to aid in the species identification of elasmobranchs from various regions, such as Australia [36,42,43], China [44], the Philippines [50], Indonesia [51], Singapore [45,47], India [19,52,53], Bangladesh [54], Southern Africa [46,55], the United Kingdom [56], the Mediterranean Sea [57], the North Atlantic Ocean [58], the United States [49], and Brazil [59,60,61]. To date, limited published DNA barcoding studies in Malaysia have focused on rays but not on sharks [9,34]. From the available records in BOLD [62], a total of 783 sequences representing 120 species (primarily from the orders Myliobatiformes and Carcharhiniformes) from Malaysian waters were found. These suggested that elasmobranchs in Malaysian waters are not completely barcoded with potential for undiscovered species. Therefore, the present study used DNA barcoding to elucidate the species diversity of sharks and batoids, as well as to update the online database of DNA sequences of elasmobranchs in Malaysian waters. The results could reveal a greater elasmobranch diversity and help support the implementation of elasmobranch conservation and management programs in Malaysian waters.

## 2. Materials and Methods

### 2.1. Tissue Sampling and Processing

**Figure 1:**
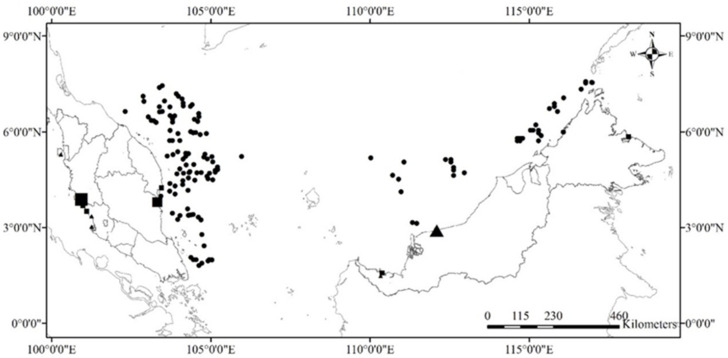
Map of the selected elasmobranch sampling sites in Malaysia.

Shark and batoid tissue samples were collected at various landing sites and markets from four major coastal areas: west coast of Peninsular Malaysia (WP), east coast of Peninsular Malaysia (EP), Sarawak (SR), and Sabah (SB) (Figure 1), from June 2015 to June 2022. These landing sites were chosen based on previous surveys conducted by the Department of Fisheries Malaysia (DOFM), which highlighted prime locations for local elasmobranch fisheries. Moreover, additional samples along EP, SR, and SB were obtained through opportunistic fisheries—independent demersal trawl surveys within the Exclusive Economic Zone (EEZ) organised by the Department of Fisheries Malaysia (July 2015 to July 2016). They were identified in the field and the laboratory by KCL and AJXL using authoritative species identification guides and taxonomic references of the region [4,6,13,14,63,64,65,66]. No live animals were collected or killed during this study. No permission to collect data from EP was needed at the time of the research, and permission to collect data from Sarawak waters was granted by the Fisheries Research Institute Sarawak, Department of Fisheries Malaysia. A Sabah sampling permit was provided by the Sabah Biodiversity Council (SaBC), Ref. No. JKM/MBS.1000-2/2 JLD.9 (22) and JKM/MBS.1000-2/3JLD.4 (18). At least one fin clip of each recorded species was collected and preserved either in absolute ethanol or gently squashed onto a Whatman FTA^®^ Elute card before the subsequent molecular analysis.

### 2.2. Extraction, PCR Amplification, and DNA Sequencing

One to six tissue samples for each species were selected for barcoding and molecular analysis (Table 1). Particular attention was given to specimens showing morphological ambiguities during field identification and potential cryptic species, as well as species that have not been barcoded to date; molecular identification was used to clarify species identities in these cases. These includes samples of *Cephaloscyllium sarawakense* (2-013 and 3-640); *Carcharhinus leucas* (3-129); *Squalus altipinnis* (2-041 and 2-047); *Narcine maculata* (2-874 and 4-439); *Rhinobatos borneensis* (KK7, 2-070, 2-678, S6, S16, and S17); *Okamejei boesemani* (2-057, 2-093, and 4-065); *Hemitrygon parvonigra* (2-059, 2-127, 2-448, and T8); *Maculabatis gerrardi* (TW5, 2-160, 2-162, 2-166, JHR5TC, and S27E); *Urogymnus lobistoma* (3-051, 3-821, and 3-876); *Myliobatis hamlyni* (T7, 2-027, and 2-029); and *Chimaera phantasma* (2-023 and 2-025). For samples stored in absolute ethanol, the total DNA was extracted using 10% Chelex resin incubated for two minutes at 60 °C, followed by 25 min at 103 °C, following a modified protocol of Hyde et al. [67]. For samples stored using Whatman FTA^®^ Elute cards, the total DNA was extracted using the modified protocol in Rigby et al. [68].

Amplification of the partial cytochrome c oxidase subunit I (CO1) gene (650 base pairs, nucleotide position 51-701) was done using universal primers FishF2– 5′ TCG ACT AAT CAT AAA GAT ATC GGC AC 3′ and FishR2—5′ ACT TCA GGG TGA CCG AAG AAT CAG AA 3′ [38]. PCR reactions were performed in 25 μL volumes containing 12.5 μL of 2X PCR Master Mix (1st Base), 1 μL of 10 mM of both primers, 1 μL DNA templates, and 9.5 μL sterilized distilled water. The thermal conditions consisted of the initial preheating at 94 °C for 5 min, denaturation at 94 °C for 30 s, annealing at 44–54 °C for 30 s, and extension at 72 °C for 1 min, then repeated for 36–40 cycles, followed by a final extension at 72 °C of 5 min. The PCR products were checked using 1% agarose in TAE buffer. Only PCR products that showed good PCR amplification were sent for sequencing service at Apical Scientific Sdn Bhd (Selangor, Malaysia). The sequencing results were checked for confirmation of the species using the BLAST tool of the NCBI and compared with field identification. The species identification matching was based on the query cover (%) in the BLAST tool as the query cover that describes how similar the sample sequence is to the reference sequence in GenBank and the similarity in the BOLD system.

### 2.3. Data Analysis

For the data analysis, the obtained sequences were edited and aligned using BioEdit version 7.2.5 [69]. Molecular species identification was achieved using two approaches: Basic Local Alignment Search Tool (BLAST^®^) and phylogenetic tree reconstruction. For the first approach, reviewed sequences were uploaded to BLAST^®^ (https://blast.ncbi.nlm.nih.gov/Blast.cgi, accessed on 5 January 2023) and BOLDSYSTEMS (http://www.boldsystems.org/index.php/IDS_OpenIdEngine, accessed on 5 January 2023) in search for local similarities with the available sequences in the National Center for Biotechnology Information (NCBI) GenBank and Barcode of Life Database (BOLD), respectively. The results from the searches were in the form of scores, query cover, and percentage identity of the 200 most similar sequences available in the databases. The list of sequences that produced significant alignments or best matches with each of our sequence was reviewed manually for validity of the suggested identity. This included their percentage similarity, query coverage, reliability of the sequence sources (from respectable authors in the field), congruency among sequences from different sources, etc. Suspicious sequences in the database were excluded in the process.

For the second approach, a phylogenetic tree was constructed using sequences from the current study and reference sequences available in the NCBI GenBank (Supplementary Materials Table S2). The selection of the reference sequences included the best matched sequences in the BLAST^®^ search, as well as available sequences of elasmobranchs that are recorded in Malaysian waters. Reference sequences from Malaysian waters were prioritized over sequences from neighbouring waters. The list of elasmobranchs (70 sharks and 91 batoid species) and chimaera species that had been recorded in Malaysia (include some unverified records) is shown in Supplementary Materials Table S1. The number of reference sequences used for each species was limited to one or two sequences, except for the species complex of *Maculabatis gerardi* and *M. macrura*.

A midpoint rooted tree maximum likelihood (ML) based on Kimura-2-Parameter (K2P) distances was created using MEGA X [70] with 1000 bootstrap replicates. The general time-reversible model plus gamma distribution rate plus evolutionarily invariable model (GTR + G + I) was selected by MEGA X as the best-fitting substitution model based on the Akaike Information Criterion (AIC). Sequences of closely related cartilaginous fish (*Chimaera phantasma*) were used as the outgroup. In this approach, morphological identification was verified based on the clustering of the samples in the phylogenetic tree in comparison to the reference sequences. Furthermore, the genetic distance was calculated to evaluate the usefulness of COI across taxonomic rank. We also examined genetic distances (*p*-distance) within and between species and subspecies. The interindividual distances calculated in MEGA X were sorted into eight inter-rank categories (intraspecific, interspecific, inter-genus, inter-subfamily, inter-family, inter-order, inter-infraclass, and inter-class) based on the species classification in the Eschmeyer’s Catalog of Fishes (CAS) [71] and the International Union for Conservation of Nature’s (IUCN) [72] Red List of Threatened Species. The mean and ranges of p-distance for these categories were calculated and boxplot was generated using Statistica 7 [73]. In addition, the analysis of variance (ANOVA) supplemented with Tukey’s HSD pairwise comparison was performed to determine the variations among inter-rank categories, between infraclass, and among orders of elasmobranchs.

## 3. Results

A total of 175 individuals belonging to 29 shark and 38 batoid species based on field identification were barcoded in this study. In addition, two individuals of chimaera *Chimaera phantasma* (closely related cartilaginous fishes) were also barcoded. All sequences were uploaded to GenBank, accession numbers: OQ384975–OQ385149 (n = 175). Another 231 sequences from NCBI GenBank were retrieved for phylogenetic tree reconstruction (Supplementary Materials Table S2). These sequences covered 82% of the total elasmobranch species recorded in Malaysia, i.e., there are 29 remaining elasmobranch species in Malaysian waters that have not been barcoded to date.

### 3.1. Matches with BLAST

Table 1 provides, for each barcoded individual, the percent matches to the best matched species in the NCBI GenBank and the similarity matched in the BOLD database. The results showed that 137 of the 175 samples strongly matched (>99%) with sequences of the same identity only (average 99.86%). The remaining individuals with uncertainty were noted as being matched with different species (13 individuals), low similarity (88.01–98.93%) with the best matched species (15 individuals), matched with multiple species at average genetic distance of 99.7% (17 individuals), or matched with unknown species (1 individual) (see Table 1).

### 3.2. Tree Matches

**Figure 2:**
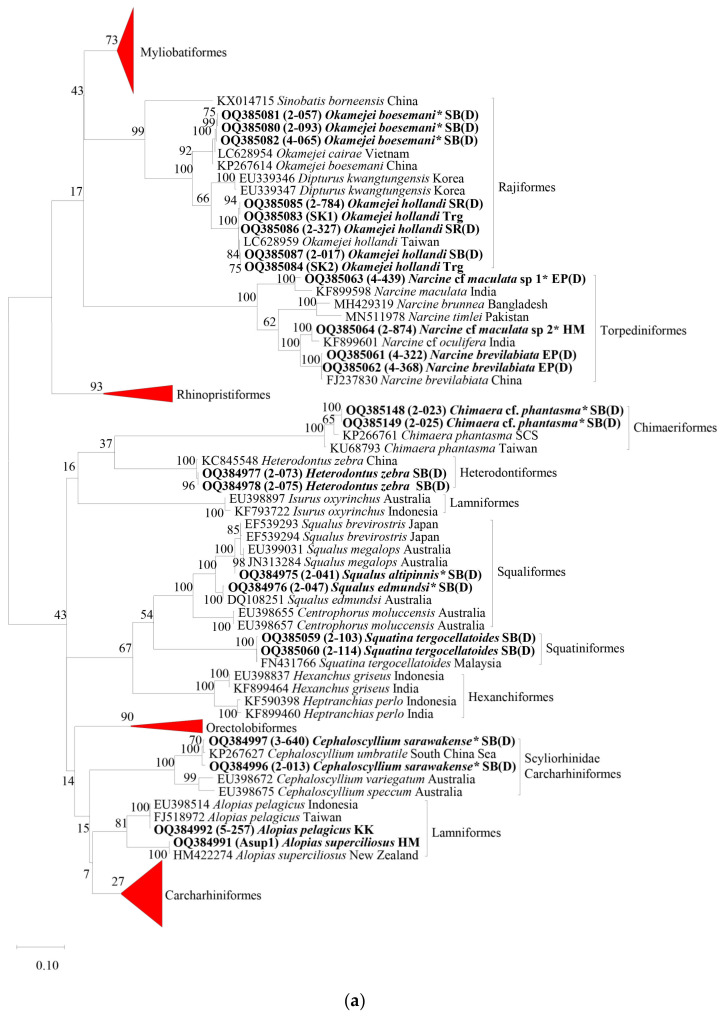

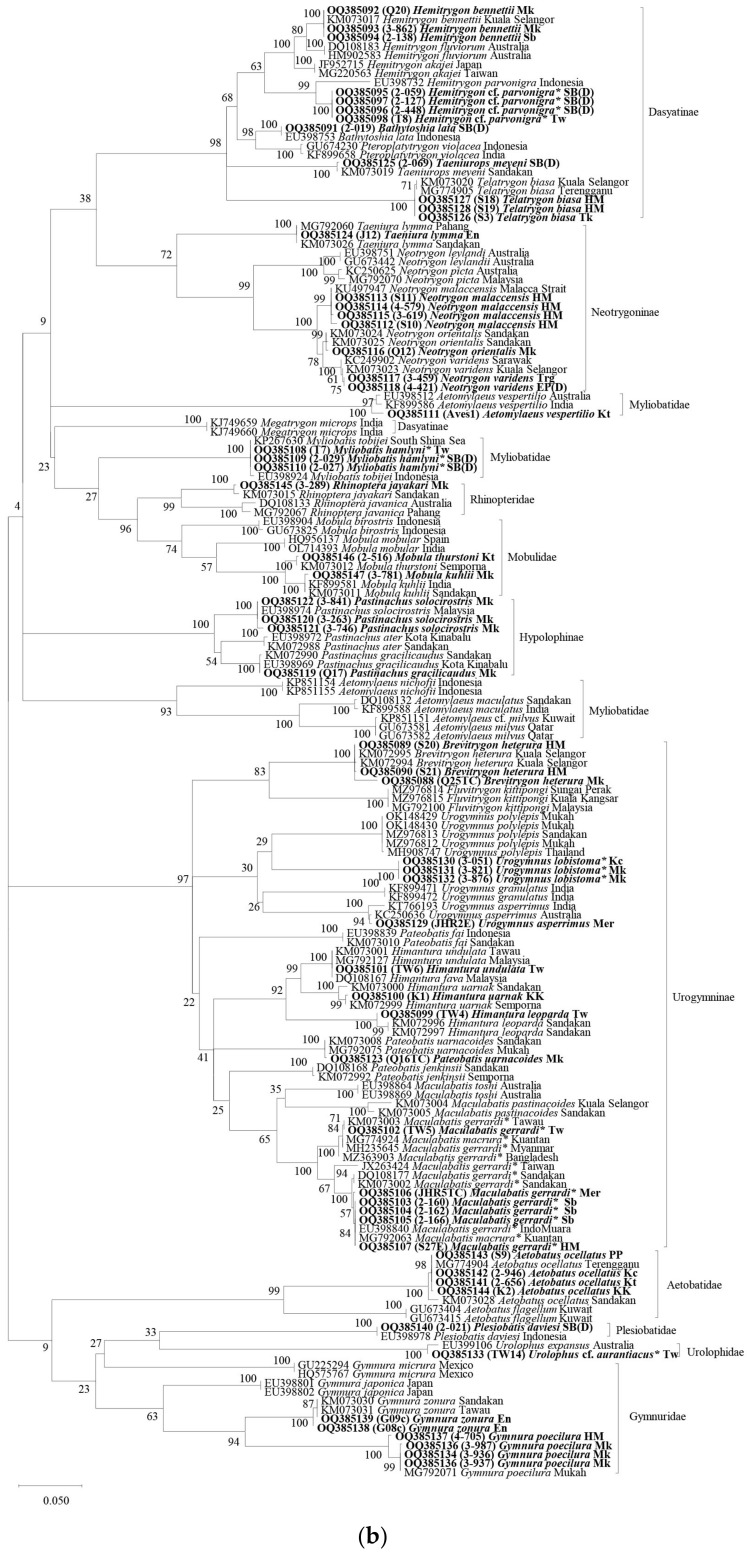

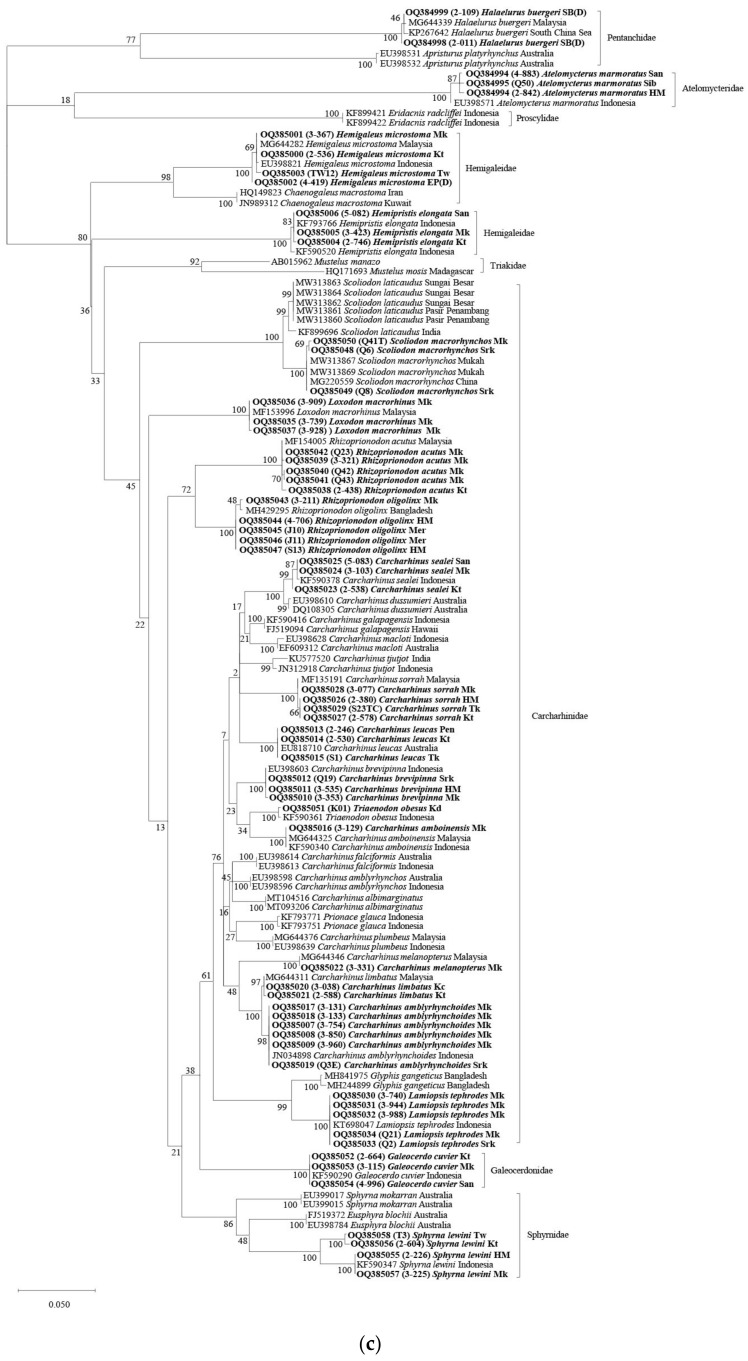

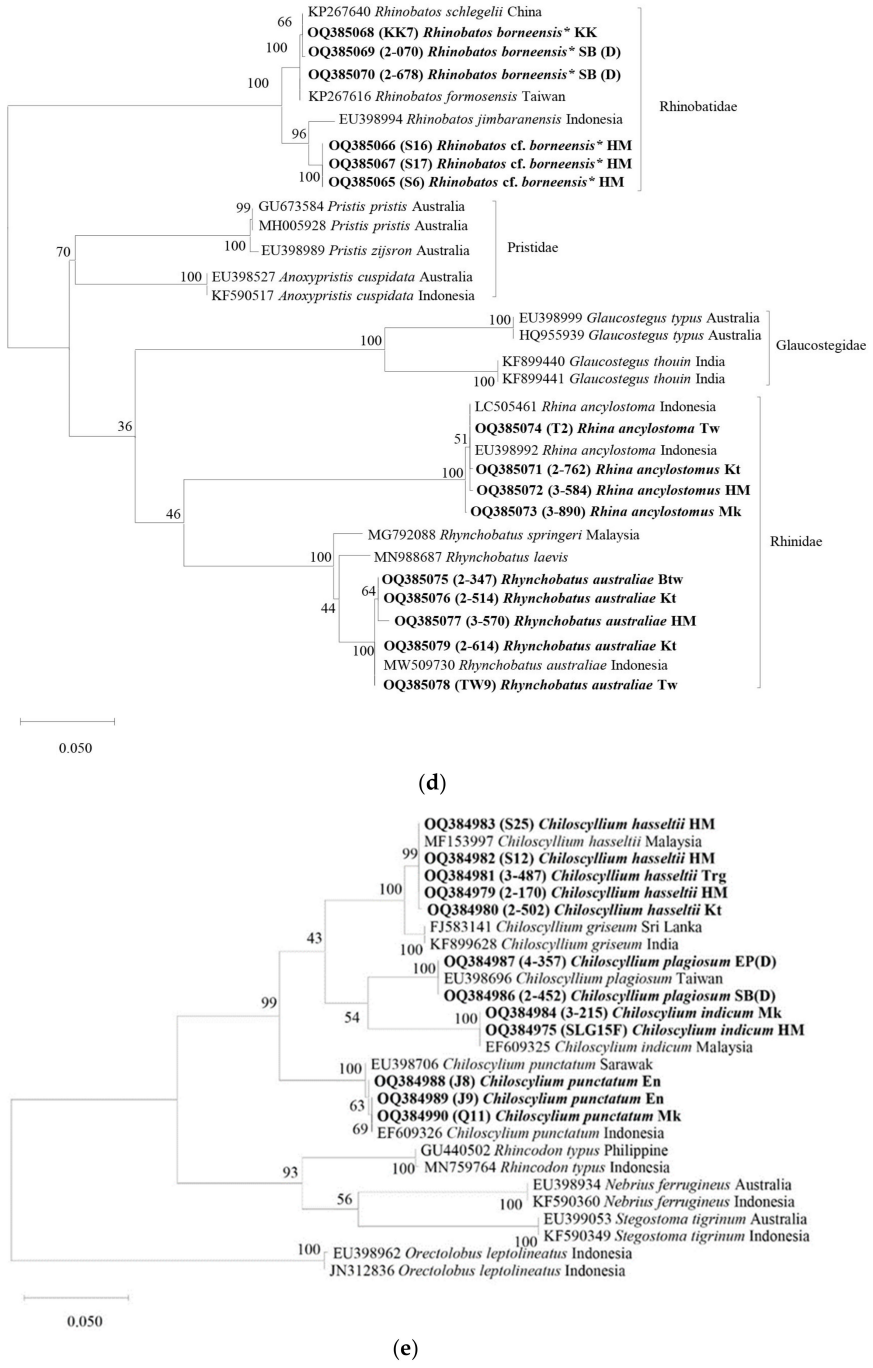
Maximum likelihood (ML) mid-point rooting tree based on Kimura-2-Parameter (K2P) distances of COI gene of (**a**) sharks and batoids. (**b**) Myliobatiformes species. (**c**) Carcharhiniformes species. (**d**) Rhinopristiformes species. (**e**) Orectolobiformes species. The bootstrap values (ML) are shown at branches. Sequence names in bold are from the present study. * Indicate specimens of concern. See Table 1 for the abbreviation of locations for sequences from the present study.

The ML tree (Figure 2a–e) matched most of the present sequences to reference sequences forming distinct clades by species (Supplementary Materials Table S3). Some of the exceptions in shark species were listed as follows. Field-identified *S. altipinnis* (samples 2-041 and 2-047) was found to be paraphyletic based on the ML tree; sample 2-041 formed a potential unique clade on its own (1.3–5.2% genetic difference with other highly similar *Squalus* species), while sample 2-047 clustered with the reference sequence of *S. edmundsi* (0.2% genetic distance). *Sphyrna lewini* (samples 2-226, 2-604, 3-225, and T3) formed two distinct clades, with sample 2-604 and T3 branching out from the other sequences, forming a unique clade (3.7–3.8% genetic distance). *Cephaloscyllium sarawakense* (samples 2-013 and 3-640) formed a clade with a reference sequence named *C. umbratile,* with genetic distance ranges from 0.6 to 1.3%. *Carcharhinus leucas* showed two separate clades: samples S1, 2-246, and 2-530 formed a clade with a reference sequence of *C. leucas*, while sample 3-129 formed a clade with a reference sequence of *C. amboinensis*. Sequences of *Carcharhinus amblyrhynchoides* and *C. limbatus* from the present study and the reference sequences used formed two separate clades; however, the genetic distance between the clades was low (0.6–0.8%).

The exceptions among batoids were listed as follows. *Hemitrygon parvonigra* (samples 2-059, 2-127, 2-448, and T8) was separated from the reference sequence of the species at 5.2% inter-clade genetic distance. The species identity of the *Neotrygon kuhlii* reference sequences found in Malaysian waters were updated to *N. malaccensis*, *N. orientalis,* and *N. varidens,* according to the assigned species names in Borsa et al. [74]. After renaming the references sequences, the *Neotrygon* sequences from the present study (samples S10, S11, Q12, 3-459, 3-619, 4-421, and 4-457) were found to cluster with the reference sequence of their respective species. *Urogymnus lobistoma* recorded in the present study showed a unique clade on its own as separate from other *Urogymnus* reference sequences (genetic distances ranged from 12.1 to 13.7%).

Sequences of *M. gerrardi* and *M. macrura* were paraphyletic showing multiple overlapping clades. The inter-individual genetic distances ranged from 0 to 5.1%. The sequence of *O. boesemani* formed a clade with reference sequences of *O. boesemani* from China (0.9–1.1% genetic distance with present sequences) and *O. cairae* from Vietnam (0.5–0.6% genetic distance with present sequences). Two *N. maculata* barcoded in this study were found to be different genetically and did not form clades with any of the best matched species; the genetic distance between samples 4-439 and *N. maculata* from India was 4.3% while that between samples 2-874 and *N.* cf. *oculifera* was 1.6%. Two or possibly three separate clades of field identified *Rhinobatos borneensis* were found with samples from Sabah (KK7, 2-070, and 2-678) forming clades with reference sequences named *R. formosensis* and *R. schlegelii* (0 to 0.3% within clade genetic distance), while samples from WP (S6, S16, and S17) were likely a unique clade separated from the aforementioned clade at 3.2 to 4.0% genetic distance and from *R. jimbaranensis* at 2.2% genetic distance. *Myliobatis hamlyni* (samples 2-027, 2-029, and T7) formed a clade with the reference sequence of the species (species name of sequence EU398924 was revised to *M. hamlyni* by White et al. [62]), as well as with *M. tobijei* sequence from South China Sea (0–0.3% genetic distance).

Although the sequences of *C. phantasma* were not the focus of this study, it was worth noting that the sequences obtained were separated from the reference sequences of *C. phantasma* at a genetic distance of 2.7 to 3.8%.

### 3.3. Final Taxonomic Identification

Considering the results of the BLAST matches and the ML tree, taxonomic identities of 121 individuals that showed consensus using both approaches were clearly verified. Seven samples of *Neotrygon* species were also confirmed after updated species identity of the reference sequences according to Borsa et al. [74]. Six samples of *C. amblyrhynchoides* (3-754, 3-850, 3-960, 3-131, 3-133, and Q3E) and two samples of *C. limbatus* (3-038 and 2-588) were cautiously treated as correctly identified due to the presence of misidentified sequences in NCBI GenBank. This suggests the need to review the identity of all submitted sequences in NCBI GenBank to prevent future confusion. Four samples of *S. lewini* (2-226, 2-604, 3-225, and T3) were also cautiously treated as correctly identified. However, the presence of two distinct clades suggested cryptic species of *S. lewini* that need further taxonomic evaluation. Three samples of *M. hamlyni* (T7, 2-027, and 2-029) were treated as correctly identified, backed by the reference sequence (EU398924) and specimens that were used in *M. hamlyni* redescription [66]. Two individuals, i.e., samples 2-047 and 3-129, were determined to be field misidentifications and were assigned as *S. edmundsi* and *C. amboinensis,* respectively, based on the phylogenetic tree information.

The identities of the remaining 30 individuals remained inconclusive based on the two molecular approaches. Field-identified *S. altipinnis* (2-041) and *U. lobistoma* (3-051, 3-821, and 3-876) were treated as the final species assignment as sequences of these species were not available in NCBI GenBank. The species identity of the two *C. sarawakense* samples was retained, as the morphological characteristics of the specimens did not match those of *C. umbratile*, suggesting a possible error in the submitted *C. umbratile* sequences in NCBI GenBank. Similarly, the identity of the *U.* cf. *aurantiacus* sample was retained, as the morphological characteristics of the sample were found to be different from the best matched species of *U. expansus*. Species identities of the sequences of three *R. borneensis* (from Sabah), three *O. boesemani,* and six *M. gerrardi* were also retained, as they belong to species complexes that need taxonomic clarification. The three other sequences of *R. borneensis* from WP were treated as *R.* cf. *borneensis* considering the low similarity with the available reference sequences of the genus. Eight sequences that matched at low similarity levels were each treated as *N.* cf. *maculata* sp1 (sample 4-439); *N.* cf. *maculata* sp2 (sample 2-874); *H.* cf. *parvonigra* (sample 2-059, 2-127, 2-448, and T8); and *C.* cf. *phantasma* (2-023 and 2-025).

### 3.4. Genetic Distance

**Figure 3:**
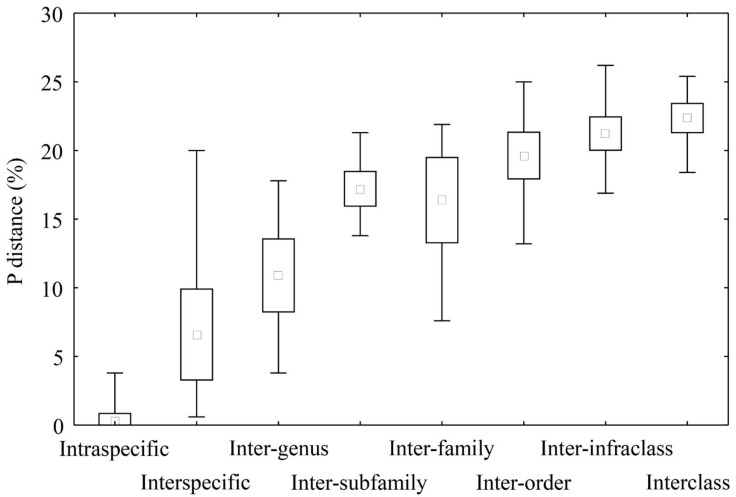
Genetic distance ranges among inter-rank categories. The middle point, box, and whiskers represent the mean, mean ± standard deviation, and total range, respectively.

Pairwise genetic distances were progressively increased as the inter-rank categories increased (Figure 3). These increases were significant (*p* < 0.001)-based overall and pairwise comparisons in the ANOVA and Tukey’s HSD tests. However, the range for among inter-rank genetic distances were considered large and overlapped especially between the neighbouring categories.

**Figure 4:**
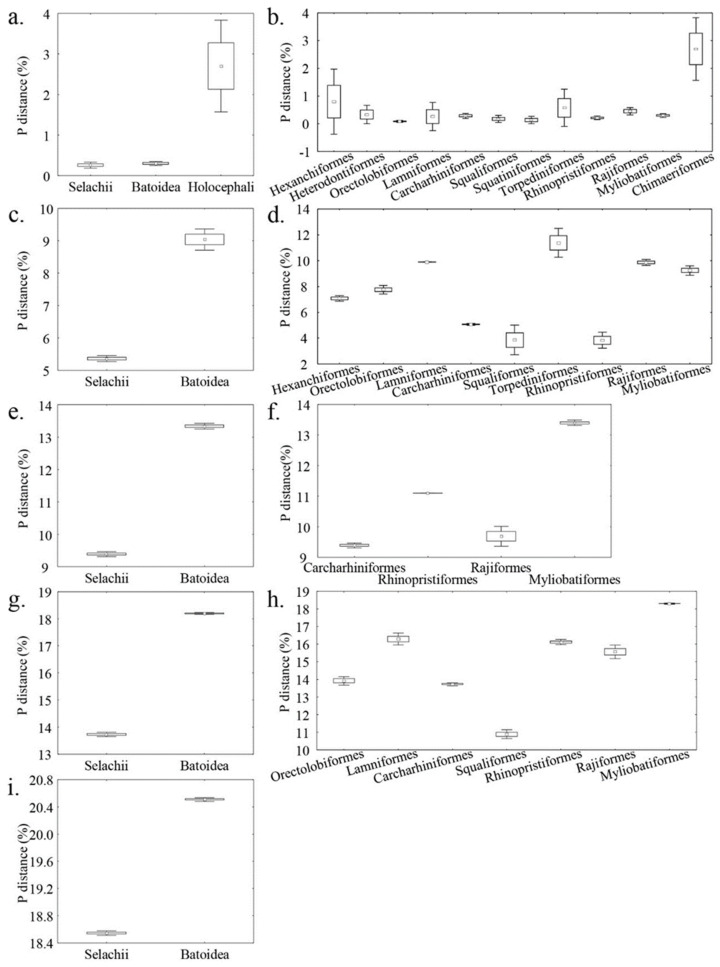
Genetic distances among infraclass (or class) (**a**,**c**,**e**,**g**,**i**) and order (**b**,**d**,**f**,**h**). (**a**,**b**) Intraspecific; (**c**,**d**) interspecific; (**e**,**f**) inter-genus; (**g**,**h**) inter-family; and (**i**) inter-order. The middle point, box, and whiskers represent the mean, mean ± standard error, and mean ± 1.96 standard error, respectively.

Comparison across class/infraclass for each of the inter-rank categories is shown in Figure 4. Within intraspecific categories, the genetic distance of Holocephali was significantly higher than the elasmobranchs (*p* < 0.001). The genetic distance within batoids, on the other hand, was significantly higher than sharks for the interspecific, inter-genus, inter-family, and inter-order categories (*p* < 0.001). Comparisons across orders provide further details for the inter-individual distances (Figure 4). Within the intraspecific categories, it showed that most of the genetic distances were in the range from 0 to 1%, except in Hexanchiformes and Chimaeriformes. For interspecific distance, the range for most batoids was found above 8%, except Rhinopristiformes, while below 8% in most of the sharks, except Lamniformes and part of the Orectolobiformes. For the inter-genus category, the genetic distance within Myliobatiformes was significantly higher than the other comparable orders (*p* < 0.001). For inter-family, the genetic distances within Myliobatiformes and Squaliformes were each highest and lowest among the comparable orders (*p* < 0.001). Other significant comparisons can be found between Orectolobiformes and Lamniformes, Orectolobiformes and Rhinopristiformes, and Carcharhiniformes with the others, except Orectolobiformes (*p* < 0.05 or *p* < 0.001).

## 4. Discussion

A comprehensive DNA barcode reference library is one of the fundamental building blocks allowing application of the increasingly popular environmental DNA (eDNA) metabarcoding approach for the assessment and environmental monitoring of biodiversity [75]. While gaps remain in the current elasmobranch biodiversity reference library, collective efforts, including ours, have advanced barcoding data for both common and rarely occurring freshwater and marine species of sharks, batoids, and skate in Malaysia. Additionally, the field identification and molecular verification efforts added four new records for Malaysia, namely *Squalus edmundsi*, *Carcharhinus amboinensis*, *Alopias superciliosus,* and *Myliobatis hamlyni*. With the exception of *A. superciliosus* that was sampled from WP, the other three species were sampled from Borneo (Sabah and Sarawak). These findings further affirm that the waters of Malaysian Borneo remain understudied in terms of the full biodiversity characterization of local elasmobranchs.

From the collation of available studies, there are 70 species of sharks (19 families) and 91 species of batoids (11 families) recorded in Malaysian waters (Supplementary Materials Table S1). The combined records from various studies had excluded species that were unaccepted in World Register of Marine Species (WORMS) (*Cephaloscyllium circulopullum* and *Narcine indica*) and species that had been verified as absent from Malaysian waters (*Brevitrygon walga* and *B. imbricata* in Last et al. [13]). These records have been improved in terms of classification within the family Dasyatidae [13]; incorporation of the revision of *Telatrygon* species in Malaysian waters [14]; delimitation of cryptic species in *Neotrygon kuhlii* complexes [15,74,76]; new recorded species in Malaysian water, including *Carcharhinus tjutjot* [10], *Fluvitrygon kittipongi,* and *F. oxyrhyncha* [16,17]); and *Scoliodon laticaudus* [35] (previously revised as *S. macrorhynchos* in Last et al. [6]).

Among the new species records, there are two species that are field-misidentified based on their morphological characteristics, namely *S. edmundsi* and *C. amboinensis*. These misidentifications are unsurprising, given how uncommon these two species are worldwide; the former was only described in 2007 in specimens from Australia [77], while the latter is likely often misidentified as highly similar-looking bull shark *C. leucas*, resulting in poor distribution records [78]. The usefulness of the molecular approach is particularly evident in separating these two morphologically similar carcharhinid species that are genetically distinctive. The discovery of spurdog *S. edmundsi* was possible due to the Department of Fisheries Malaysia trawl survey within the deeper EEZ waters off the coast of Borneo. This relatively newly described species is currently known to occur only off the continental waters of Australia and Indonesia [79].

The new finding of the bigeye thresher *A. superciliosus* in a major fisheries landing site in Hutan Melintang, along the central-west coast of Peninsula Malaysia, was rather surprising. This thresher species is thought to occur in deeper waters and generally less catchable than the tropical congener *A. pelagicus* [80], which had only been reported off the open waters of Sabah. The trawl fisheries in the landing site where A. supercilious was found typically operates in the relatively shallow Strait of Malacca. Further investigations into this finding will help shed light on the ecology of the thresher.

For the eagle ray *M. hamlyni*, existing molecular evidence suggested a best match with *M. tobijei* from South China Sea (0% genetic difference) and *M. hamlyni* from Indonesia (0.3% genetic distance). The latest distributional assessment on both species by White et al. [66] showed that *M. tobijei* is restricted to the western North Pacific, while *M. hamlyni* occurs in neighbouring Indonesia and the Philippines. Moreover, the morphological characteristic of the present specimen best matches the species description of *M. hamlyni* in White et al. [66], especially the purplish brown dorsal coloration (compared to yellowish brown in *M. tobijei*), as well as the slightly convex cranial fontanelle that is visible in the dorsal view (nearly straight and broader in *M. tobijei*). More importantly, the *M. hamlyni* specimen corresponded to the reference sequence used in the present analysis (EU398924), i.e., one of the type of specimens in White et al. [66]. This suggests that the reference sequence for *M. tobijei* KP267630 (published in December 2015) was likely an outdated species identity. Prior to the White et al. [66] redescription study, *M. tobijei* was thought to be distributed from Japan down south to Indonesia and the Philippines [66]. The field-identified *U.* cf. *aurantiacus* showed the highest genetic similarity to *U. expansus* found only in Southwestern Australia, but the known distribution of *U. aurantiacus* in the Philippines makes the latter species identity more feasible. The *U.* cf. *aurantiacus* specimen may therefore be an undescribed species, and the significance of this finding is that no urolophids are known to occur within Malaysia to date.

One of the most fundamental requirements in conservation and fishery management of sharks and batoids is correct species identification. While some advances have been made from our study, improvement in the records is still needed, including verification of the 40 recorded species of concern (recorded by single/multiple reference/s by the same research team or absent in Malaysia according to the IUCN assessment). The use of available genetic sequences without careful morphological examination and corroboration from existing distributional records would result in erroneous species assignment. The BOLD species assignment of *Narcine* cf. *oculifera* and *Cephaloscyllium umbratile* are inconclusive, as the percentage match with the present sequences (except sample 3-640) was less than 99%, and they had not been previously recorded in Malaysian waters. Despite the use of multiple approaches, our work highlights the need to focus on select taxonomic groups that may be species complexes or cryptic species. *Sphyrna lewini* (which was also pointed out by Naylor et al. [81]), the *R. borneensis-schlegelli* group, and the *M. gerrardi-macrura* group are among those requiring further investigations that are feasible due to the relatively common occurrence locally from our past surveys. On the other hand, taxonomic work to resolve unclear identities of uncommon species of *S. altipinnis* (2-041); *C. sarawakense* (2-013 and 3-064); *N. maculata* (4-439 and 2-874); and *H. parvonigra* (2-059, 2-448, 2-127, and T8) may be hampered without extensive field samplings.

The use of barcoding approaches is also fundamental in describing species that are still unknown to the scientific communities. This is important to prevent the loss of species before even being discovered, as seen in *Carcharhinus obsolerus* [82]. However, to advance the use of barcoding for accurate biodiversity monitoring, there remains the urgent need for the scientific names in the NCBI database to be updated regularly. One example is the revision of the family Dasyatidae that has resulted in the renaming of *Himantura walga* to *Brevitrygon heterura* (the revised *B. walga* is found to be confined to the Indian Ocean) [13]. Another example is the identity of reference sequence EU398924 as *M. tobijei* in NCBI GenBank, which likely should be *M. hamlyni* after the updated study of White et al. [66]. It is imperative to review the identity of all submitted sequences in NCBI GenBank to prevent future confusion, preferably through collaboration with reputable taxonomist with molecular knowledge to further enhance the validity of the submitted sequences. Moving forward, it is also essential to increase the amount of localized data on individual species, and the correct identification of species through traditional morphology and the uploading of correct sequences for the right species are highly essential to allow full functionality of barcoding technology for species identification.

## 5. Conclusions

The barcoding of animal tissues has proven to be an important technique to identify species from animal remains that cannot be identified by conventional morphological means and therefore cannot be adequately traced. Accurate species identification will improve species-specific catch landing data and ease and accelerate the identification of their illegal trade and use in Malaysia. Overall, this will provide baseline and monitoring data for conservation and fisheries management. The sharks and batoids CO1 database establishment and improvement will also help facilitate the rapid monitoring and assessment of elasmobranch fish through environmental DNA methods. In the future, rapid and large-scale surveys can be conducted to obtain the distribution points of cartilaginous fishes and predict their potential habitat areas, thereby providing a scientific basis and basis for the identification of elasmobranch conservation priority areas.

## Figures and Tables

**Table 1:** Verification of the identity of barcoded shark and batoids species. Given are the sample code, sample location, NCBI accession number, field identification, best matched species from NCBI GenBank, similarity species and % from BOLD, tree-based identification, and the consensus identification. West coast of Peninsular Malaysia (WP): HM = Hutan Melintang, Pen = Penang, PP = Pasir Penambang, Sb = Sungai Besar, and Tk = Tanjung Karang; East coast of Peninsular Malaysia (EP): En = Endau, EP(D) = East coast of Peninsular Malaysia (DOF survey), Kt = Kuantan, Mer = Mersing, and Trg = Terengganu; Sarawak (SR): Btw = Bintawa, Kc = Kuching, Mk = Mukah, Sib = Sibu, SR(D) = Sarawak (DOF survey), and Srk = Sarikei; Sabah (SB): Kd = Kudat, KK = Kota Kinabalu, San = Sandakan, SB(D) = Sabah (DOF survey), and Tw = Tawau.

Samples	Location	Accession Number	Field Identification	NCBI Best Matched	%	BOLD Similarity	%	Tree Match	Consensus Identification
2-073	SB(D)	OQ384975	*Heterodontus zebra*	*H. zebra*	99.54	*H. zebra*	100	*H. zebra*	*H. zebra*
2-075	SB(D)	OQ384976	*Heterodontus zebra*	*H. zebra*	99.54	*H. zebra*	100	*H. zebra*	*H. zebra*
2-170	HM	OQ384977	*Chiloscyllium hasseltii*	*C. hasseltii*	100	*C. hasseltii/griseum*	100	*C. hasseltii*	*C. hasseltii*
2-502	Kt	OQ384978	*Chiloscyllium hasseltii*	*C. hasseltii*	100	*C. hasseltii griseum*	99.85	*C. hasseltii*	*C. hasseltii*
3-487	Trg	OQ384979	*Chiloscyllium hasseltii*	*C. hasseltii*	100	*C. hasseltii griseum*	100	*C. hasseltii*	*C. hasseltii*
S12	HM	OQ384980	*Chiloscyllium hasseltii*	*C. hasseltii*	100	*C. hasseltii griseum*	100	*C. hasseltii*	*C. hasseltii*
S25	HM	OQ384981	*Chiloscyllium hasseltii*	*C. hasseltii*	100	*C. hasseltii griseum*	100	*C. hasseltii*	*C. hasseltii*
3-215	Mk	OQ384982	*Chiloscyllium indicum*	*C. indicum*	100	*C. indicum*	100	*C. indicum*	*C. indicum*
SLG15	HM	OQ384983	*Chiloscyllium indicum*	*C. indicum*	100	*C. indicum*	100	*C. indicum*	*C. indicum*
2-452	SB(D)	OQ384984	*Chiloscyllium plagiosum*	*C. plagiosum*	99.85	*C. plagiosum*	100	*C. plagiosum*	*C. plagiosum*
4-357	EP(D)	OQ384985	*Chiloscyllium plagiosum*	*C. plagiosum*	99.85	*C. plagiosum*	100	*C. plagiosum*	*C. plagiosum*
J8	En	OQ384986	*Chiloscyllium punctatum*	*C. punctatum*	99.69	*C. punctatum*	99.85	*C. punctatum*	*C. punctatum*
J9	En	OQ384987	*Chiloscyllium punctatum*	*C. punctatum*	99.69	*C. punctatum*	100	*C. punctatum*	*C. punctatum*
Q11	Mk	OQ384988	*Chiloscyllium punctatum*	*C. punctatum*	99.69	*C. punctatum*	100	*C. punctatum*	*C. punctatum*
5-257	KK	OQ384989	*Alopias pelagicus*	*A. pelagicus*	100	*A. pelagicus*	100	*A. pelagicus*	*A. pelagicus*
Asup1	HM	OQ384990	*Alopias superciliosus*	*A. superciliosus*	100	*A. superciliosus*	100	*A. superciliosus*	*A. superciliosus*
2-842	HM	OQ384991	*Atelomycterus marmoratus*	*A. marmoratus*	100	*A. marmoratus*	100	*A. marmoratus*	*A. marmoratus*
4-883	San	OQ384992	*Atelomycterus marmoratus*	*A. marmoratus*	99.53	*A. marmoratus*	99.84	*A. marmoratus*	*A. marmoratus*
Q50	Sib	OQ384993	*Atelomycterus marmoratus*	*A. marmoratus*	99.37	*A. marmoratus*	99.85	*A. marmoratus*	*A. marmoratus*
2-013	SB(D)	OQ384994	*Cephaloscyllium sarawakense*	*C. umbratile* ^LS^	98.93	*C. umbratile* ^LS^	98.92	-	*C. sarawakense* ^I^
3-640	SB(D)	OQ384995	*Cephaloscyllium sarawakense*	*C. umbratile* ^DS^	99.85	*C. umbratile* ^DS^	99.85	*-*	*C. sarawakense* ^I^
2-011	SB(D)	OQ384996	*Halaelurus buergeri*	*H. buergeri*	99.69	*H. buergeri*	99.85	*H. buergeri*	*H. buergeri*
2-109	SB(D)	OQ384997	*Halaelurus buergeri*	*H. buergeri*	99.69	*H. buergeri*	99.85	*H. buergeri*	*H. buergeri*
2-536	Kt	OQ384998	*Hemigaleus microstoma*	*H. microstoma*	100	*H. microstoma*	100	*H. microstoma*	*H. microstoma*
3-367	Mk	OQ384999	*Hemigaleus microstoma*	*H. microstoma*	100	*H. microstoma*	100	*H. microstoma*	*H. microstoma*
4-419	EP(D)	OQ385000	*Hemigaleus microstoma*	*H. microstoma*	100	*H. microstoma*	100	*H. microstoma*	*H. microstoma*
TW12	Tw	OQ385001	*Hemigaleus microstoma*	*H. microstoma*	99.69	*H. microstoma*	100	*H. microstoma*	*H. microstoma*
2-746	Kt	OQ385002	*Hemipristis elongata*	*H. elongata*	100	*H. elongata*	100	*H. elongata*	*H. elongata*
3-423	Mk	OQ385003	*Hemipristis elongata*	*H. elongata*	100	*H. elongata*	100	*H. elongata*	*H. elongata*
5-082	San	OQ385004	*Hemipristis elongata*	*H. elongata*	99.69	*H. elongata*	100	*H. elongata*	*H. elongata*
3-754	Mk	OQ385005	*Carcharhinus amblyrhynchoides*	*C. amblyrhynchoides* ^MM^	100	*C. amblyrhynchoides* ^MM^	100	*C. amblyrhynchoides*	*C. amblyrhynchoides*
3-850	Mk	OQ385006	*Carcharhinus amblyrhynchoides*	*C. amblyrhynchoides* ^MM^	100	*C. amblyrhynchoides* ^MM^	100	*C. amblyrhynchoides*	*C. amblyrhynchoides*
3-960	Mk	OQ385007	*Carcharhinus amblyrhynchoides*	*C. amblyrhynchoides* ^MM^	100	*C. amblyrhynchoides* ^MM^	100	*C. amblyrhynchoides*	*C. amblyrhynchoides*
3-131	Mk	OQ385008	*Carcharhinus amblyrhynchoides*	*C. amblyrhynchoides* ^MM^	100	*C. amblyrhynchoides* ^MM^	100	*C. amblyrhynchoides*	*C. amblyrhynchoides*
3-133	Mk	OQ385009	*Carcharhinus amblyrhynchoides*	*C. amblyrhynchoides* ^MM^	100	*C. amblyrhynchoides* ^MM^	100	*C. amblyrhynchoides*	*C. amblyrhynchoides*
Q3E	Srk	OQ385010	*Carcharhinus amblyrhynchoides*	*C. amblyrhynchoides* ^MM^	100	*C. amblyrhynchoides* ^MM^	100	*C. amblyrhynchoides*	*C. amblyrhynchoides*
3-353	Mk	OQ385011	*Carcharhinus brevipinna*	*C. brevipinna*	100	*C. brevipinna*	99.85	*C. brevipinna*	*C. brevipinna*
3-535	HM	OQ385012	*Carcharhinus brevipinna*	*C. brevipinna*	100	*C. brevipinna*	100	*C. brevipinna*	*C. brevipinna*
Q19	Srk	OQ385013	*Carcharhinus brevipinna*	*C. brevipinna*	100	*C. brevipinna*	100	*C. brevipinna*	*C. brevipinna*
2-246	Pen	OQ385014	*Carcharhinus leucas*	*C. leucas*	100	*C. leucas*	100	*C. leucas*	*C. leucas*
2-530	Kt	OQ385015	*Carcharhinus leucas*	*C. leucas*	100	*C. leucas*	100	*C. leucas*	*C. leucas*
S1	Tk	OQ385016	*Carcharhinus leucas*	*C. leucas*	100	*C. leucas*	100	*C. leucas*	*C. leucas*
3-129	Mk	OQ385017	*Carcharhinus leucas*	*C. amboinensis* ^DS^	99.69	*C. amboinensis* ^DS^	100	*C. amboinensis*	*C. amboinensis*
3-038	Kc	OQ385018	*Carcharhinus limbatus*	*C. limbatus* ^MM^	100	*C. limbatus* ^MM^	100	*C. limbatus*	*C. limbatus*
2-588	Kt	OQ385019	*Carcharhinus limbatus*	*C. limbatus* ^MM^	99.54	*C. limbatus* ^MM^	99.84	*C. limbatus*	*C. limbatus*
3-331	Mk	OQ385020	*Carcharhinus melanopterus*	*C. melanopterus*	100	*C. melanopterus*	100	*C. melanopterus*	*C. melanopterus*
2-538	Kt	OQ385021	*Carcharhinus sealei*	*C. sealei*	100	*C. sealei*	100	*C. sealei*	*C. sealei*
3-103	Mk	OQ385022	*Carcharhinus sealei*	*C. sealei*	99.85	*C. sealei*	100	*C. sealei*	*C. sealei*
5-083	San	OQ385023	*Carcharhinus sealei*	*C. sealei*	99.85	*C. sealei*	100	*C. sealei*	*C. sealei*
2-380	HM	OQ385024	*Carcharhinus sorrah*	*C. sorrah*	99.85	*C. sorrah*	99.85	*C. sorrah*	*C. sorrah*
2-578	Kt	OQ385025	*Carcharhinus sorrah*	*C. sorrah*	99.85	*C. sorrah*	99.85	*C. sorrah*	*C. sorrah*
3-077	Mk	OQ385026	*Carcharhinus sorrah*	*C. sorrah*	100	*C. sorrah*	100	*C. sorrah*	*C. sorrah*
S23TC	Tk	OQ385027	*Carcharhinus sorrah*	*C. sorrah*	99.85	*C. sorrah*	99.85	*C. sorrah*	*C. sorrah*
3-740	Mk	OQ385028	*Lamiopsis tephrodes*	*L. tephrodes*	100	*L. tephrodes*	100	*L. tephrodes*	*L. tephrodes*
3-944	Mk	OQ385029	*Lamiopsis tephrodes*	*L. tephrodes*	100	*L. tephrodes*	100	*L. tephrodes*	*L. tephrodes*
3-988	Mk	OQ385030	*Lamiopsis tephrodes*	*L. tephrodes*	100	*L. tephrodes*	100	*L. tephrodes*	*L. tephrodes*
Q2	Srk	OQ385031	*Lamiopsis tephrodes*	*L. tephrodes*	100	*L. tephrodes*	100	*L. tephrodes*	*L. tephrodes*
Q21	Mk	OQ385032	*Lamiopsis tephrodes*	*L. tephrodes*	100	*L. tephrodes*	100	*L. tephrodes*	*L. tephrodes*
3-739	Mk	OQ385033	*Loxodon macrorhinus*	*L. macrorhinus*	100	*L. macrorhinus*	100	*L. macrorhinus*	*L. macrorhinus*
3-909	Mk	OQ385034	*Loxodon macrorhinus*	*L. macrorhinus*	100	*L. macrorhinus*	100	*L. macrorhinus*	*L. macrorhinus*
3-928	Mk	OQ385035	*Loxodon macrorhinus*	*L. macrorhinus*	99.85	*L. macrorhinus*	99.85	*L. macrorhinus*	*L. macrorhinus*
2-438	Kt	OQ385036	*Rhizoprionodon acutus*	*R. acutus*	100	*R. acutus*	100	*R. acutus*	*R. acutus*
3-321	Mk	OQ385037	*Rhizoprionodon acutus*	*R. acutus*	99.85	*R. acutus*	99.85	*R. acutus*	*R. acutus*
Q42	Mk	OQ385038	*Rhizoprionodon acutus*	*R. acutus*	99.85	*R. acutus*	99.85	*R. acutus*	*R. acutus*
Q43	Mk	OQ385039	*Rhizoprionodon acutus*	*R. acutus*	99.85	*R. acutus*	99.85	*R. acutus*	*R. acutus*
Q23	Mk	OQ385040	*Rhizoprionodon acutus*	*R. acutus*	100	*R. acutus*	100	*R. acutus*	*R. acutus*
3-211	Mk	OQ385041	*Rhizoprionodon oligolinx*	*R. oligolinx*	99.69	*R. oligolinx*	99.85	*R. oligolinx*	*R. oligolinx*
4-706	HM	OQ385042	*Rhizoprionodon oligolinx*	*R. oligolinx*	99.54	*R. oligolinx*	100	*R. oligolinx*	*R. oligolinx*
J10	Mer	OQ385043	*Rhizoprionodon oligolinx*	*R. oligolinx*	99.54	*R. oligolinx*	100	*R. oligolinx*	*R. oligolinx*
J11	Mer	OQ385044	*Rhizoprionodon oligolinx*	*R. oligolinx*	99.54	*R. oligolinx*	100	*R. oligolinx*	*R. oligolinx*
S13	HM	OQ385045	*Rhizoprionodon oligolinx*	*R. oligolinx*	99.54	*R. oligolinx*	100	*R. oligolinx*	*R. oligolinx*
Q6	Srk	OQ385046	*Scoliodon macrorhynchos*	*S. macrorhynchos*	100	*S. macrorhynchos*	100	*S. macrorhynchos*	*S. macrorhynchos*
Q8	Srk	OQ385047	*Scoliodon macrorhynchos*	*S. macrorhynchos*	100	*S. macrorhynchos*	100	*S. macrorhynchos*	*S. macrorhynchos*
Q41T	Mk	OQ385048	*Scoliodon macrorhynchos*	*S. macrorhynchos*	99.85	*S. macrorhynchos*	99.85	*S. macrorhynchos*	*S. macrorhynchos*
K01	Kd	OQ385049	*Triaenodon obesus*	*T. obesus*	99.69	*T. obesus*	100	*T. obesus*	*T. obesus*
2-664	Kt	OQ385050	*Galeocerdo cuvier*	*G. cuvier*	100	*G. cuvier*	100	*G. cuvier*	*G. cuvier*
3-115	Mk	OQ385051	*Galeocerdo cuvier*	*G. cuvier*	100	*G. cuvier*	100	*G. cuvier*	*G. cuvier*
4-996	San	OQ385052	*Galeocerdo cuvier*	*G. cuvier*	100	*G. cuvier*	100	*G. cuvier*	*G. cuvier*
2-226	HM	OQ385053	*Sphyrna lewini*	*S. lewini*	100	*S. lewini*	100	*S. lewini*	*S. lewini*
2-604	Kt	OQ385054	*Sphyrna lewini*	*S. lewini*	99.69	*S. lewini*	99.80	*S. lewini*	*S. lewini*
3-225	Mk	OQ385055	*Sphyrna lewini*	*S. lewini*	100	*S. lewini*	100	*S. lewini*	*S. lewini*
T3	Tw	OQ385056	*Sphyrna lewini*	*S. lewini*	100	*S. lewini*	100	*S. lewini*	*S. lewini*
2-041	SB(D)	OQ385057	*Squalus altipinnis*	*S. brevirostris* ^LS^	98.78	*S. brevirostris* ^LS^	98.92	-	*S. altipinnis* ^I^
2-047	SB(D)	OQ385058	*Squalus altipinnis*	*S. edmundsi* ^DS^	99.54	*S. edmundsi* ^DS^	100	*S. edmundsi*	*S. edmundsi*
2-103	SB(D)	OQ385059	*Squatina tergocellatoides*	*S. tergocellatoides*	99.85	*S. tergocellatoides*	99.85	*S. tergocellatoides*	*S. tergocellatoides*
2-114	SB(D)	OQ385060	*Squatina tergocellatoides*	*S. tergocellatoides*	100	*S. tergocellatoides*	100	*S. tergocellatoides*	*S. tergocellatoides*
4-322	EP(D)	OQ385061	*Narcine brevilabiata*	*N. brevilabiata*	99.69	*N. brevilabiata*	99.85	*N. brevilabiata*	*N. brevilabiata*
4-368	EP(D)	OQ385062	*Narcine brevilabiata*	*N. brevilabiata*	99.85	*N. brevilabiata*	100	*N. brevilabiata*	*N. brevilabiata*
4-439	EP(D)	OQ385063	*Narcine maculata*	*N. maculata* ^LS^	95.25	*N. maculata* ^LS^	98.52	*-*	*N.* cf. *maculata* sp. 1 ^I^
2-874	HM	OQ385064	*Narcine maculata*	*Narcine* sp.^MU^	99.35	*Narcine* cf. *oculifera* ^MU^	99.69	-	*N.* cf. *maculata* sp. 2 ^I^
2-762	Kt	OQ385065	*Rhina ancylostomus*	*R. ancylostomus*	99.85	*R. ancylostomus*	99.83	*R. ancylostomus*	*R. ancylostomus*
3-584	HM	OQ385066	*Rhina ancylostomus*	*R. ancylostomus*	100	*R. ancylostomus*	99.85	*R. ancylostomus*	*R. ancylostomus*
3-890	Mk	OQ385067	*Rhina ancylostomus*	*R. ancylostomus*	99.69	*R. ancylostomus*	99.67	*R. ancylostomus*	*R. ancylostomus*
T2	Tw	OQ385068	*Rhina ancylostomus*	*R. ancylostomus*	100	*R. ancylostomus*	100	*R. ancylostomus*	*R. ancylostomus*
2-347	Btw	OQ385069	*Rhynchobatus australiae*	*R. australiae*	100	*R. australiae*	100	*R. australiae*	*R. australiae*
2-514	Kt	OQ385070	*Rhynchobatus australiae*	*R. australiae*	100	*R. australiae*	100	*R. australiae*	*R. australiae*
3-570	HM	OQ385071	*Rhynchobatus australiae*	*R. australiae*	99.39	*R. australiae*	99.62	*R. australiae*	*R. australiae*
TW9	Tw	OQ385072	*Rhynchobatus australiae*	*R. australiae*	100	*R. australiae*	100	*R. australiae*	*R. australiae*
2-614	Kt	OQ385073	*Rhynchobatus australiae*	*R. australiae*	100	*R. australiae*	100	*R. australiae*	*R. australiae*
KK7	KK	OQ385074	*Rhinobatos borneensis*	*R. schlegelii* ^DS^	100	*R. schlegelii* ^DS^	100	-	*R. borneensis* ^I^
2-070	SB(D)	OQ385075	*Rhinobatos borneensis*	*R. schlegelii* ^DS^	99.85	*R. schlegelii* ^DS^	99.85	-	*R. borneensis* ^I^
2-678	SB(D)	OQ385076	*Rhinobatos borneensis*	*R. formosensis* ^DS^	100	*R. formosensis* ^DS^	99.85	-	*R. borneensis* ^I^
S6	HM	OQ385077	*Rhinobatos* cf *borneensis*	*R. jimbaranensis* ^LS^	97.54	*R. jimbaranensis* ^LS^	97.64	-	*R.* cf. *borneensis* ^I^
S16	HM	OQ385078	*Rhinobatos* cf *borneensis*	*R. jimbaranensis* ^LS^	97.54	*R. jimbaranensis* ^LS^	97.64	-	*R.* cf. *borneensis* ^I^
S17	HM	OQ385079	*Rhinobatos* cf *borneensis*	*R. jimbaranensis* ^LS^	97.54	*R. jimbaranensis* ^LS^	97.64	-	*R.* cf. *borneensis* ^I^
2-093	SB(D)	OQ385080	*Okamejei boesemani*	*O. boesemani* ^MM^	99.38	*O. boesemani* ^MM^	99.38	-	*O. boesemani* ^I^
2-057	SB(D)	OQ385081	*Okamejei boesemani*	*O. boesemani* ^MM^	99.38	*O. boesemani* ^MM^	99.38	-	*O. boesemani* ^I^
4-065	SR(D)	OQ385082	*Okamejei boesemani*	*O. boesemani* ^MM^	99.54	*O. boesemani* ^MM^	99.53	-	*O. boesemani* ^I^
SK1	Trg	OQ385083	*Okamejei hollandi*	*O. hollandi*	99.52	*O. hollandi*	99.51	*O. hollandi*	*O. hollandi*
SK2	Trg	OQ385084	*Okamejei hollandi*	*O. hollandi*	99.84	*O. hollandi*	99.84	*O. hollandi*	*O. hollandi*
2-784	SR(D)	OQ385085	*Okamejei hollandi*	*O. hollandi*	99.52	*O. hollandi*	99.51	*O. hollandi*	*O. hollandi*
2-327	SR(D)	OQ385086	*Okamejei hollandi*	*O. hollandi*	99.68	*O. hollandi*	99.68	*O. hollandi*	*O. hollandi*
2-017	SB(D)	OQ385087	*Okamejei hollandi*	*O. hollandi*	99.84	*O. hollandi*	99.84	*O. hollandi*	*O. hollandi*
2-019	SB(D)	OQ385088	*Bathytoshia lata*	*B. lata*	100	*B. lata*	100	*B. lata*	*B. lata*
Q25TC	Mk	OQ385089	*Brevitrygon heterura*	*B. heterura*	99.69	*B. heterura*	99.69	*B. heterura*	*B. heterura*
S20	HM	OQ385090	*Brevitrygon heterura*	*B. heterura*	100	*B. heterura*	100	*B. heterura*	*B. heterura*
S21	HM	OQ385091	*Brevitrygon heterura*	*B. heterura*	99.85	*B. heterura*	99.85	*B. heterura*	*B. heterura*
Q20	Mk	OQ385092	*Hemitrygon bennettii*	*H. bennettii*	100	*H. bennettii*	100	*H. bennettii*	*H. bennettii*
3-862	Mk	OQ385093	*Hemitrygon bennettii*	*H. bennettii*	100	*H. bennettii*	100	*H. bennettii*	*H. bennettii*
2-138	Sb	OQ385094	*Hemitrygon bennettii*	*H. bennettii*	100	*H. bennettii*	100	*H. bennettii*	*H. bennettii*
2-059	SB(D)	OQ385095	*Hemitrygon parvonigra*	*H. parvonigra* ^LS^	94.78	*H. fai* ^LS^	100	-	*H.* cf. *parvonigra* ^I^
2-448	SB(D)	OQ385096	*Hemitrygon parvonigra*	*H. parvonigra* ^LS^	94.78	*H. fai* ^LS^	100	-	*H.* cf. *parvonigra* ^I^
2-127	SB(D)	OQ385097	*Hemitrygon parvonigra*	*H. parvonigra* ^LS^	94.78	*H. fai* ^LS^	100	-	*H.* cf. *parvonigra* ^I^
T8	Tw	OQ385098	*Hemitrygon parvonigra*	*H. parvonigra* ^LS^	94.78	*H. fai* ^LS^	100	-	*H.* cf. *parvonigra* ^I^
TW4	Tw	OQ385099	*Himantura leoparda*	*H. leoparda*	99.08	*H. leoparda*	99.19	*H. leoparda*	*H. leoparda*
K1	KK	OQ385100	*Himantura uarnak*	*H. uarnak*	100	*H. uarnak*	100	*H. uarnak*	*H. uarnak*
TW6	Tw	OQ385101	*Himantura undulata*	*H. undulata*	100	*H. undulata*	100	*H. undulata*	*H. undulata*
TW5	Tw	OQ385102	*Maculabatis gerrardi*	*M. macrura* ^MM^	99.85	*M. macrura/H. gerrardi* ^MM^	100	*M. gerrardi*	*M. gerrardi* ^I^
2-160	Sb	OQ385103	*Maculabatis* *gerrardi*	*M. macrura/H. gerrardi* ^MM^	100	*M. macrura/H. gerrardi* ^MM^	100	*M. gerrardi*	*M. gerrardi* ^I^
2-162	Sb	OQ385104	*Maculabatis gerrardi*	*M. macrura/H. gerrardi* ^MM^	100	*M. macrura/H. gerrardi* ^MM^	100	*M. gerrardi*	*M. gerrardi* ^I^
2-166	Sb	OQ385105	*Maculabatis gerrardi*	*M. macrura/H. gerrardi* ^MM^	100	*M. macrura/H. gerrardi* ^MM^	100	*M. gerrardi*	*M. gerrardi* ^I^
JHR5TC	Mer	OQ385106	*Maculabatis gerrardi*	*M. macrura/H. gerrardi* ^MM^	99.69	*M. macrura/H. gerrardi* ^MM^	100	*M. gerrardi*	*M. gerrardi* ^I^
S27E	HM	OQ385107	*Maculabatis gerrardi*	*M. macrura/H. gerrardi* ^MM^	100	*M. macrura/H. gerrardi* ^MM^	100	*M. gerrardi*	*M. gerrardi* ^I^
S10	HM	OQ385108	*Neotrygon malaccensis*	*N. kuhlii* ^DS^	99.54	*N. kuhlii* ^DS^	99.84	*N. malaccensis*	*N. malaccensis*
S11	HM	OQ385109	*Neotrygon malaccensis*	*N. kuhlii* ^DS^	100	*N. kuhlii* ^DS^	100	*N. malaccensis*	*N. malaccensis*
3-619	HM	OQ385110	*Neotrygon malaccensis*	*N. kuhlii* ^DS^	99.85	*N. kuhlii* ^DS^	100	*N. malaccensis*	*N. malaccensis*
4-579	HM	OQ385111	*Neotrygon malaccensis*	*N. kuhlii* ^DS^	100	*N. kuhlii* ^DS^	100	*N. malaccensis*	*N. malaccensis*
Q12	Mk	OQ385112	*Neotrygon orientalis*	*N. kuhlii* ^DS^	100	*N. kuhlii* ^DS^	100	*N. orientalis*	*N. orientalis*
3-459	Trg	OQ385113	*Neotrygon varidens*	*N. kuhlii* ^DS^	100	*N. kuhlii* ^DS^	100	*N. varidens*	*N. varidens*
4-421	EP(D)	OQ385114	*Neotrygon varidens*	*N. kuhlii* ^DS^	99.85	*N. kuhlii* ^DS^	100	*N. varidens*	*N. varidens*
Q17	Mk	OQ385115	*Pastinachus gracilicaudus*	*P. gracilicaudus*	100	*P. gracilicaudus/* *solocirostris*	100	*P. gracilicaudus*	*P. gracilicaudus*
3-263	Mk	OQ385116	*Pastinachus solocirostris*	*P. solocirostris*	100	*P. solocirostris*	100	*P. solocirostris*	*P. solocirostris*
3-746	Mk	OQ385117	*Pastinachus solocirostris*	*P. solocirostris*	100	*P. solocirostris*	100	*P. solocirostris*	*P. solocirostris*
3-841	Mk	OQ385118	*Pastinachus solocirostris*	*P. solocirostris*	100	*P. solocirostris*	100	*P. solocirostris*	*P. solocirostris*
Q16TC	Mk	OQ385119	*Pateobatis uarnacoides*	*P. uarnacoides*	99.85	*P. uarnacoides*	100	*P. uarnacoides*	*P. uarnacoides*
J12	En	OQ385120	*Taeniura lymma*	*T. lymma*	100	*T. lymma*	100	*T. lymma*	*T. lymma*
2-069	SB(D)	OQ385121	*Taeniurops meyeni*	*T. meyeni*	99.85	*T. meyeni*	100	*T. meyeni*	*T. meyeni*
S3	Tk	OQ385122	*Telatrygon biasa*	*T. biasa*	99.69	*T. zugei*	100	*T. biasa*	*T. biasa*
S18	HM	OQ385123	*Telatrygon biasa*	*T. biasa*	99.54	*T. zugei*	99.83	*T. biasa*	*T. biasa*
S19	HM	OQ385124	*Telatrygon biasa*	*T. biasa*	99.54	*T. zugei*	99.83	*T. biasa*	*T. biasa*
JHR2E	Mer	OQ385125	*Urogymnus asperrimus*	*U. asperrimus*	99.69	*U. asperrimus*	99.85	*U. asperrimus*	*U. asperrimus*
3-051	Kc	OQ385126	*Urogymnus lobistoma*	*H. chaophraya*	88.01	*H. uarnacoides*	100	*-*	*U. lobistoma* ^I^
3-821	Mk	OQ385127	*Urogymnus lobistoma*	*H. chaophraya*	88.01	*H. uarmacoides*	100	-	*U. lobistoma* ^I^
3-876	Mk	OQ385128	*Urogymnus lobistoma*	*H. chaophraya*	88.01	*H. uarmacoides*	100	-	*U. lobistoma* ^I^
3-936	Mk	OQ385129	*Gymnura poecilura*	*G. poecilura*	100	*G. poecilura*	100	*G. poecilura*	*G. poecilura*
3-937	Mk	OQ385130	*Gymnura poecilura*	*G. poecilura*	100	*G. poecilura*	100	*G. poecilura*	*G. poecilura*
3-987	Mk	OQ385131	*Gymnura poecilura*	*G. poecilura*	99.85	*G. poecilura*	99.84	*G. poecilura*	*G. poecilura*
4-705	HM	OQ385132	*Gymnura poecilura*	*G. poecilura*	99.69	*G. poecilura*	99.69	*G. poecilura*	*G. poecilura*
G08c	En	OQ385133	*Gymnura zonura*	*G. zonura*	99.54	*G. zonura*	99.69	*G. zonura*	*G. zonura*
G09c	En	OQ385134	*Gymnura zonura*	*G. zonura*	99.85	*G. zonura*	100	*G. zonura*	*G. zonura*
2-021	SB(D)	OQ385135	*Plesiobatis daviesi*	*P. daviesi*	100	*P. daviesi*	100	*P. daviesi*	*P. daviesi*
TW14	Tw	OQ385136	*Urolophus* cf. *aurantiacus*	*U. expansus* ^DS^	99.07	*U. expansus* ^DS^	99.84	-	*U.* cf. *aurantiacus* ^I^
2-656	Kt	OQ385137	*Aetobatus ocellatus*	*A. ocellatus*	100	*A. ocellatus*	100	*A. ocellatus*	*A. ocellatus*
2-946	Kc	OQ385138	*Aetobatus ocellatus*	*A. ocellatus*	100	*A. ocellatus*	100	*A. ocellatus*	*A. ocellatus*
S9	PP	OQ385139	*Aetobatus ocellatus*	*A. ocellatus*	100	*A. ocellatus*	100	*A. ocellatus*	*A. ocellatus*
K2	KK	OQ385140	*Aetobatus ocellatus*	*A. ocellatus*	99.85	*A. ocellatus*	99.85	*A. ocellatus*	*A. ocellatus*
Aves1	Kt	OQ385141	*Aetomylaeus vespertilio*	*A vespertilio*	99.07	*A vespertilio*	98.88	*A vespertilio*	*A vespertilio*
T7	Tw	OQ385142	*Myliobatis hamlyni*	*M. hamlyni ^R^*	100	*M. tobijei*	100	*M. tobijei*	*M. hamlyni*
2-027	SB(D)	OQ385143	*Myliobatis hamlyni*	*M. hamlyni ^R^*	100	*M. tobijei*	100	*M. tobijei*	*M. hamlyni*
2-029	SB(D)	OQ385144	*Myliobatis hamlyni*	*M. hamlyni ^R^*	100	*M. tobijei*	100	*M. tobijei*	*M. hamlyni*
3-289	Mk	OQ385145	*Rhinoptera jayakari*	*R. jayakari*	100	*R. jayakari*	100	*R. jayakari*	*R. jayakari*
2-516	Kt	OQ385146	*Mobula thurstoni*	*M. thurstoni*	100	*M. thurstoni*	100	*M. thurstoni*	*M. thurstoni*
3-781	Mk	OQ385147	*Mobula kuhlii*	*M. kuhlii*	100	*M. kuhlii*	100	*M. kuhlii*	*M. kuhlii*
2-023	SB(D)	OQ385148	*Chimaera phantasma*	*C. phantasma* ^LS^	97.39	*C. phantasma* ^LS^	97.67	-	*C.* cf. *phantasma* ^I^
2-025	SB(D)	OQ385149	*Chimaera phantasma*	*C. phantasma* ^LS^	97.39	*C. phantasma* ^LS^	97.67	-	*C.* cf. *phantasma* ^I^

^R^ = Revised identity based on White et al. [66]—reference sequence EU398924 *M. tobijei* was revised as *M. hamlyni*; ^DS^ = best match differed from field-identified species; ^LS^ = best match showed low similarity; ^MM^ = match with multiple species; ^MU^ = match with uncertain species; and ^I^ = inconclusive identity.

## Data Availability

All sequence (n = 175) results are uploaded to GenBank, accession numbers: OQ384975–OQ385149.

## Supplementary Materials

The following supporting information can be downloaded at: https://www.mdpi.com/article/10.3390/ani13061002/s1: Table S1: Compilation of elasmobranch records in Malaysia waters, latest IUCN status, and COI sequence availability in NCBI GenBank. Summary represent inferences based on available records. Table S2: The 231 references sequences from NCBI GenBank were retrieved for phylogenetic tree reconstruction. Table S3: Data for the estimates of evolutionary divergence between sequences.
