# Supplementary material for: Advancing DNA Barcoding to Elucidate Elasmobranch Biodiversity in Malaysian Waters
Source: Animals (Basel). 2023 Mar 9;13(6):1002. doi: 10.3390/ani13061002 (PMC10044685; doi:10.3390/ani13061002)
Supplement: Supplementary file 1 [file animals-13-01002-s001.zip › animals-2241340-supplementary.pdf]

Supplementary Materials S1. Compilation of elasmobranch records in Malaysian waters, latest IUCN status, and COI sequence availability in NCBI Genbank. Summary represent inferences based on available records.

Species	IUCN	NCBI	Summary	1	2	3	4	5	6	7	8	9	10	Other
**Order: Hexanchiformes**														
**Family Hexanchidae**														
Hexanchus griseus	NT^{2020}	Y	Y			Y	Y	Y	Y					
Heptranchias perlo	NT^{2020}	Y	Y*						Y					
**Order: Heterodontiformes**														
**Family: Heterodontidae**														
Heterodontus zebra	LC^{2020}	Y	Y		Y	Y	Y	Y	Y			Y		
**Order: Orectolobiformes**														
**Family: Rhincodontidae**														
Rhincodon typus	EN^{2016}	Y	Y		Y	Y	Y	Y	Y				Y	
**Family: Orectolobidae**														
Orectolobus leptolineatus	NT^{2021}	Y	Y			Y	Y	Y	Y			Y	Y	
**Family: Hemiscylliidae**														
Chiloscyllium griseum	VU^{2020}	Y	Y*			Y	Y		Y					
Chiloscyllium hasseltii	EN^{2020}	Y	Y			Y	Y	Y	Y			Y	Y	
Chiloscyllium indicum	VU^{2020}	Y	Y		Y	Y	Y	Y	Y			Y	Y	
Chiloscyllium plagiosum	NT^{2021}	Y	Y	Y		Y	Y	Y	Y			Y	Y	
Chiloscyllium punctatum	NT^{2016}	Y	Y		Y	Y	Y	Y	Y			Y	Y	
**Family: Stegostomatidae**														
Stegostoma tigrinum	EN^{2019}	Y	Y	Y	Y	Y	Y	Y	Y				Y	
**Family: Ginglymostomatidae**														
Nebrius ferrugineus	VU^{2021}	Y	Y	Y		Y	Y	Y	Y					
**Order: Lamniformes**														
**Family: Alopiidae**														
Alopias pelagicus	EN^{2019}	Y	Y			Y	Y	Y	Y			Y	Y	
**Family: Lamnidae**														
Isurus oxyrinchus	EN^{2019}	Y	Y			Y	Y	Y	Y					
**Order: Carcharhiniformes**														
**Family: Scyliorhinidae**														
Cephaloscyllium circulopullum	-	-	N^{U}			Y	Y		Y					
Cephaloscyllium sarawakense	CR^{2021}	N	Y			Y	Y	Y	Y					
Cephaloscyllium cf. speccum	DD^{2016}	Y	Y*^{I}						Y					
Cephaloscyllium cf. variegatum	NT^{2019}	Y	Y*^{I}						Y					
**Family: Atelomycteridae**														
Atelomycterus erdmanni	LC^{2021}	Y	Y*^{I}										Y	
Atelomycterus marmoratus	NT^{2021}	Y	Y	Y	Y	Y	Y	Y	Y			Y		
**Family: Pentanchidae**														
Apristurus platyrhynchus	LC^{2015}	Y	Y					Y	Y					
Galeus cf. eastmani	LC^{2020}	N	Y*^{I}						Y					
Halaelurus buergeri	EN^{2020}	Y	Y			Y	Y		Y			Y		
Halaelurus maculosus	NT^{2020}	N	Y					Y	Y					
**Family: Proscylliidae**														
Eridacnis cf. radcliffei	LC^{2019}	Y	Y*						Y					
**Family: Triakidae**														
Mustelus manazo	EN^{2020}	Y	Y			Y	Y	Y	Y				Y	
Mustelus mosis	NT^{2019}	Y	Y*^{I}			Y	Y		Y					
Mustelus sp.	-	N	Y*			Y	Y		Y					
Mustelus widodoi	VU^{2021}	N	Y					Y	Y			Y		
**Family: Hemigaleidae**														
Chaenogaleus macrostoma	VU^{2021}	Y	Y			Y	Y	Y	Y					
Hemigaleus microstoma	VU^{2021}	Y	Y		Y	Y	Y	Y	Y			Y	Y	
Hemipristis elongata	VU^{2016}	Y	Y			Y	Y	Y	Y			Y	Y	
Paragaleus tengi	EN^{2020}	N	Y			Y	Y	Y	Y					
**Family: Carcharhinidae**														
Carcharhinus albimarginatus	VU^{2021}	Y	Y					Y	Y					
Carcharhinus amblyrhynchoides	VU^{2021}	Y	Y			Y	Y	Y	Y			Y	Y	
Carcharhinus amblyrhynchos	EN^{2020}	Y	Y			Y		Y	Y					
Carcharhinus borneensis	CR^{2021}	N	Y			Y	Y	Y	Y					
Carcharhinus brevipinna	VU^{2020}	Y	Y			Y	Y	Y	Y			Y	Y	
Carcharhinus dussumieri	EN^{2019}	Y	Y^{I}		Y	Y	Y	Y	Y					
Carcharhinus falciformis	VU^{2021}	Y	Y		Y	Y	Y	Y	Y				Y	
Carcharhinus galapagensis	LC^{2019}	Y	Y*^{I}			Y	Y							
Carcharhinus hemiodon	CR^{2021}	N	Y*			Y	Y							
Carcharhinus leucas	VU^{2021}	Y	Y			Y	Y	Y	Y			Y	Y	
Carcharhinus limbatus	VU^{2021}	Y	Y		Y	Y	Y	Y	Y			Y	Y	
Carcharhinus macloti	NT^{2021}	Y	Y		Y	Y	Y	Y	Y					
Carcharhinus melanopterus	VU^{2020}	Y	Y	Y	Y	Y	Y	Y	Y			Y	Y	
Carcharhinus plumbeus	EN^{2021}	Y	Y			Y	Y	Y	Y			Y	Y	
Carcharhinus sealei	VU^{2021}	Y	Y		Y	Y	Y	Y	Y			Y	Y	

Carcharhinus sorrah	NT[2021]	Y	Y		Y	Y	Y	Y	Y			Y	Y	
Carcharhinus tjutjot	VU[2019]	Y	Y									Y		White[11]
Carcharhinus sp.	-	N	Y*			Y	Y							
Glyphis sp.	-	N	Y					Y	Y					
Glyphis gangeticus	CR[2021]	Y	Y			Y	Y	Y	Y					
Lamiopsis tephrodes	EN[2021]	Y	Y			Y	Y	Y	Y				Y	
Loxodon macrorhinus	NT[2021]	Y	Y			Y	Y	Y	Y			Y	Y	
Prionace glauca	NT[2019]	Y	Y					Y	Y					
Rhizoprionodon acutus	VU[2020]	Y	Y	Y	Y	Y	Y	Y	Y			Y	Y	
Rhizoprionodon oligolinx	NT[2021]	Y	Y			Y	Y	Y	Y				Y	
Scoliodon laticaudus	NT[2021]	Y	Y		Y	Y	Y							
Scoliodon macrorhynchos	NT[2020]	Y	Y					Y	Y			Y	Y	
Triaenodon obesus	VU[2020]	Y	Y			Y	Y	Y	Y			Y	Y	
**Family: Galeocerdonidae**														
Galeocerdo cuvier	NT[2019]	Y	Y			Y	Y	Y	Y			Y	Y	
**Family: Sphyrnidae**														
Eusphyra blochii	EN[2016]	Y	Y	Y		Y	Y	Y	Y					
Sphyrna lewini	CR[2019]	Y	Y		Y	Y	Y	Y	Y			Y	Y	
Sphyrna mokarran	CR[2019]	Y	Y		Y	Y	Y	Y	Y				Y	
Sphyrna zygaena	VU[2019]	Y	Y[*I]	Y	Y									
**Order: Squaliformes**														
**Family: Centrophoridae**														
Centrophorus moluccensis	VU[2020]	Y	Y			Y	Y	Y	Y					
**Family: Squalidae**														
Squalus altipinnis	DD[2019]	N	Y					Y	Y			Y		
Squalus japonicus	EN[2020]	Y	Y[*I]		Y									
Squalus megalops	LC[2020]	Y	Y[I]			Y	Y		Y					
**Order: Squatiniformes**														
**Family: Squatinidae**														
Squatina tergocellatoides	EN[2020]	Y	Y			Y	Y	Y	Y					
**Order: Torpediniformes**														
**Family: Narkidae**														
Narke dipterygia	VU[2021]	N	Y	Y				Y		Y				
Temera hardwickii	VU[2021]	N	Y	Y	Y			Y		Y				
**Family: Narcinidae**														
Narcine brevilabiata	VU[2020]	Y	Y					Y		Y				
Narcine brunnea	-	Y	Y*			Y				Y				
Narcine indica	-	-	N[U]	Y		Y				Y				
Narcine lingula	VU[2020]	N	Y					Y		Y				
Narcine maculata	VU[2020]	Y	Y			Y		Y		Y				
Narcine prodorsalis	EN[2021]	N	Y[*I]			Y				Y				
Narcine timlei	VU[2021]	Y	Y*		Y	Y				Y				
Narcine sp. D	-	N	Y*							Y				
**Order: Rhinopristiformes**														
**Family: Rhinobatidae**														
Rhinobatos borneensis	EN[2021]	N[P]	Y			Y		Y		Y			Y	
**Family: Rhinidae**														
Rhina ancylostoma	CR[2019]	Y	Y	Y		Y		Y		Y			Y	
Rhynchobatus australiae	CR[2019]	Y	Y			Y		Y		Y			Y	
Rhynchobatus djiddensis	CR[2019]	Y	Y[*I]	Y	Y									
Rhynchobatus laevis	CR[2019]	Y	Y[I]			Y		Y		Y			Y	
Rhynchobatus springeri	CR[2019]	Y	Y					Y		Y			Y	
**Family: Glaucostegidae**														
Glaucostegus halavi	CR[2019]	N	Y[*I]							Y				
Glaucostegus thouin	CR[2021]	Y	Y	Y	Y	Y		Y		Y			Y	
Glaucostegus typus	CR[2019]	Y	Y			Y		Y		Y			Y	
**Family: Pristidae**														
Anoxypristis cuspidata	EN[2013]	Y	Y	Y	Y			Y		Y				
Pristis microdon	-	N	Y*			Y		Y						
Pristis pristis	CR[2013]	Y	Y*							Y				
Pristis zijsron	CR[2013]	Y	Y			Y		Y		Y				
**Order: Rajiformes**														
**Family: Rajidae**														
Dipturus kwangtungensis	DD[2020]	Y	Y					Y		Y				
Okamejei boesemani	VU[2020]	Y	Y[I]			Y								
Okamejei cairae	VU[2020]	N	Y					Y		Y			Y	
Okamejei hollandi	VU[2020]	Y	Y		Y	Y		Y		Y				
Orbiraja jensenae	NT[2020]	N	Y					Y		Y				
**Family: Anacanthobatidae**														
Sinobatis borneensis	LC[2020]	Y	Y					Y		Y				
**Order: Myliobatiformes**														
**Family: Dasyatidae**														
**Subfamily: Dasyatinae**														
Bathytoshia cf lata	-	N	Y*			Y				Y				
Bathytoshia lata	VU[2021]	Y	Y*					Y						

Hemitrygon akajei	NT^{2021}	Y	Y*^{I}			Y				Y			Y	
Hemitrygon bennetti	VU^{2020}	Y	Y							Y	Y		Y	
Hemitrygon fluviorum	NT^{2021}	Y	Y*^{I}			Y				Y				
Hemitrygon parvonigra	DD^{2016}	Y	Y					Y		Y				
Hemitrygon sinensis	EN^{2020}	N	Y*^{I}			Y				Y				
Megatrygon microps	DD^{2016}	Y	Y		Y			Y		Y				
Pteroplatytrygon violacea	LC^{2019}	Y	Y			Y		Y		Y				
Taeniurops meyeni	VU^{2015}	Y	Y		Y	Y		Y		Y	Y			
Telatrygon biasa^{D}	VU^{2021}	Y	Y											Last[12]
Telatrygon cf acutirostra	VU^{2021}	N	Y*^{I}							Y				
Telatrygon zugei	VU^{2021}	Y	N^{D}	Y	Y	Y		Y		Y	Y		Y	
**Subfamily: Neotrygoninae**														
Neotrygon cf leylandii	LC^{2015}	Y	Y*							Y				
Neotrygon cf picta	LC^{2015}	Y	Y*							Y				
Neotrygon kuhlii	DD^{2018}	Y	N		Y	Y								
Neotrygon malaccensis	-	Y	Y							Y	Y			Borsa[14]
Neotrygon orientalis	LC^{2021}	Y	Y					Y		Y	Y		Y	Borsa[14]
Neotrygon varidens	LC^{2021}	Y	Y							Y				Borsa[14]
Taeniura lymma	LC^{2021}	Y	Y	Y	Y	Y		Y		Y	Y		Y	
**Subfamily: Urogymninae**														
Brevitrygon heterura	VU^{2021}	Y	Y								Y		Y	Last[13]
Brevitrygon imbricata	VU^{2021}	N	N^{L}	Y	Y	Y				Y			Y	
Brevitrygon walga	NT^{2017}	N	N^{L}		Y	Y		Y		Y				
Fluvitrygon kittipongi	EN^{2021}	Y	Y											Lim[16]
Fluvitrygon oxyrhyncha	EN^{2021}	N	Y											Hasan[15]
Fluvitrygon cf signifer	-	N	Y*							Y				
Fluvitrygon signifer	EN^{2021}	N	Y*			Y		Y						
Himantura fava	-	Y	Y*							Y				
Himantura leoparda	VU^{2016}	Y	Y					Y		Y	Y		Y	
Himantura uarnak	EN^{2021}	Y	Y	Y	Y	Y		Y		Y	Y		Y	
Himantura undulata	EN^{2020}	Y	Y			Y		Y		Y	Y		Y	
Maculabatis astra	LC^{2016}	N	Y*^{I}							Y				
Maculabatis gerrardi	EN^{2020}	Y	Y			Y		Y		Y	Y		Y	
Maculabatis macrura	EN^{2020}	Y	Y*										Y	
Maculabatis pastinacoides	EN^{2020}	Y	Y					Y		Y	Y		Y	
Maculabatis toshi	LC^{2016}	Y	Y*^{I}			Y				Y				
Pateobatis bleekeri	EN^{2020}	N	Y*							Y				
Pateobatis fai	VU^{2016}	Y	Y			Y		Y		Y	Y			
Pateobatis jenkinsii	VU^{2020}	Y	Y			Y		Y		Y	Y		Y	
Pateobatis uarnacoides	EN^{2020}	Y	Y			Y		Y		Y	Y		Y	
Urogymnus asperrimus	VU^{2016}	Y	Y	Y		Y		Y		Y				
Urogymnus granulatus	VU^{2020}	Y	Y			Y		Y		Y				
Urogymnus lobistoma	EN^{2020}	N	Y					Y		Y			Y	
Urogymnus polylepis	EN^{2021}	Y	Y			Y		Y		Y			Y	
**Subfamily: Hypolophinae**														
Pastinachus ater	VU^{2021}	Y	Y					Y		Y	Y		Y	
Pastinachus gracilicaudus	EN^{2021}	Y	Y					Y		Y	Y		Y	
Pastinachus sephen	NT^{2017}	Y	N	Y	Y	Y								
Pastinachus solocirostris	EN^{2021}	Y	Y					Y		Y			Y	
**Family: Gymnuridae**														
Gymnura japonica	VU^{2021}	Y	Y*^{I}			Y				Y			Y	
Gymnura micrura	NT^{2021}	Y	Y*^{I}	Y						Y				
Gymnura poecilura	VU^{2021}	Y	Y		Y	Y		Y		Y			Y	
Gymnura zonura	EN^{2021}	Y	Y		Y	Y		Y		Y	Y		Y	
**Family: Plesiobatidae**														
Plesiobatis daviesi	LC^{2015}	Y	Y			Y		Y		Y				
**Family: Aetobatidae**														
Aetobatus flagellum	EN^{2021}	Y	Y					Y		Y			Y	
Aetobatus narinari	EN^{2021}	Y	N	Y	Y	Y								
Aetobatus ocellatus	VU^{2016}	Y	Y					Y		Y	Y		Y	
**Family: Myliobatidae**														
Aetomylaeus maculatus	EN^{2020}	Y	Y					Y		Y				
Aetomylaeus milvus	EN^{2017}	Y	Y*^{I}	Y						Y				
Aetomylaeus nichofii	VU^{2016}	Y	Y	Y		Y		Y		Y				
Aetomylaeus vespertilio	EN^{2016}	Y	Y			Y		Y		Y			Y	
**Family: Rhinopteridae**														
Rhinoptera adspersa	-	N	Y*	Y		Y				Y				
Rhinoptera javanica	EN^{2021}	Y	Y		Y	Y		Y		Y	Y		Y	
Rhinoptera jayakari	EN^{2021}	Y	Y					Y		Y	Y		Y	
**Family: Mobulidae**														
Manta alfredi	VU^{2019}	N	Y*							Y				
Manta birostris	EN^{2020}	Y	Y*			Y				Y				
Mobula sp.	-	N	Y					Y		Y				
Mobula eregoodootenkee	EN^{2020}	-	N^{U}	Y						Y				
Mobular mobular	EN^{2020}	Y	Y		Y	Y		Y		Y			Y	

Mobula kuhlii	EN^{2020}	Y	Y			Y		Y		Y	Y			
Mobula thurstoni	EN^{2019}	Y	Y					Y		Y	Y		Y	
**Order: Chimaeriformes**														
**Family: Chimaeridae**														
Chimaera phantasma	VU^{2020}	Y	Y					Y		Y				

1: Cantor[2]; 2: Mohsin & Ambak[3]; 3: Yano et al.[4]; 4: Ahmad et al.[5]; 5: Last et al.[6]; 6: Ahmad & Lim[7]; 7: Ahmad et al.[8]; 8: Lim et al.[9]; 9: Arai & Azri[10]; 10: Booth et al.[11]; 11: White[12]; 12: Last et al.[13]; 13: Last et al.[14]; 14: Borsa et al.[15]; 15: Hasan et al.[16]; 16: Lim et al.[17]

* uncertain/unverified record, ^{I} not in Malaysia according to IUCN assessment, ^{U} unaccepted in World Register of Marine Species (WORMS), ^{L} Not in Malaysian according to Last et al. [13], ^{D} Not in Malaysia according to Last et al.[14]

Supplementary Materials Table S2. The 231 references sequences from NCBI GenBank were retrieved for phylogenetic tree reconstruction.

Species	Location	Accession	Submission year	Seq code
Hexanchus griseus	India, Kochi	KF899464	2014	KF899464_Hgris_Ind
Hexanchus griseus	Indonesia, Nusa tenggara barat	EU398837	2016	EU398837_Hgris_Indo
Heptranchias perlo	India, Kochi	KF899460	2014	KF899460_Hperl_Ind
Heptranchias perlo	Indonesia: Perlabuhan ratu west java	KF590398	2015	KF590398_Hperl_Indo
Squalus brevirostris	Japan: Kyushu-chiho	EF539293	2012	EF539293_Sbrev_JP
Squalus brevirostris	Japan: Kyushu-chiho	EF539294	2012	EF539294_Sbrev_JP
Squalus megalops	Australia, western	EU399031	2012	EU399031_Smega_Aus
Squalus megalops	Australia, western	JN313284	2019	JN313284_Smega_Aus
Squalus edmundsi	Australia, western	DQ108251	2012	DQ108251_Sedmu_Aus
Centrophorus moluccensis	Australia, western	EU398655	2012	EU398655_Cmolu_Aus
Centrophorus moluccensis	Indonesia, Nusa tenggara barat	EU398657	2016	EU398657_Cmolu_Aus
Squatina tergocellatoides	Sabah: Malaysia	FN431766	2016	FN431766_Sterg_MY
Heterodontus zebra	China	KC845548	2014	KC845548_Hzebr_Chi
Orectolobus leptolineatus	Indonesia, Nusa tenggara barat	JN312836	2019	JN312836_Olept_Indo
Orectolobus leptolineatus	Indonesia, Nusa tenggara barat	EU398962	2016	EU398962_Olept_Indo
Chiloscyllium griseum	Sri Lanka	FJ583141	2012	FJ583141_Cgris_SriL
Chiloscyllium griseum	India, Kochi	KF899628	2014	KF899628_Cgris_Ind
Chiloscyllium hasseltii	Malaysia	MF153997	2017	MF153997_Chas_My
Chiloscyllium indicum	Malaysia, Mukah	EF609325	2016	EF609325_Cind_My
Chiloscyllium plagiosum	Taiwan	EU398696	2016	EU398696_Cpla_TW
Chiloscyllium punctatum	Indonesia	EF609326	2016	EF609326_Cpun_Indo
Chiloscyllium punctatum	Malaysia, Mukah	EU398706	2016	EU398706_Cpun_MySar
Nebrius ferrugineus	Indonesia, South Sulawesi	KF590360	2015	KF590360_Nferr_Indo
Nebrius ferrugineus	Australia, western	EU398934	2012	EU398934_Nferr_Aus
Stegostoma tigrinum	Indonesia, Aceh	KF590349	2015	KF590349_Sfasc_Indo
Stegostoma tigrinum	Australia, Queensland	EU399053	2012	EU399053_Sfasc_Aus
Rhincodon typus	Philippines	GU440502	2016	GU440502_Rtypu_Phi
Rhincodon typus	Indonesia, Papua	MN759764	2020	MN759764_Rtypu_Indo
Alopias pelagicus	Indonesia, Nusa tenggara barat	EU398514	2016	EU398514_Apela_Indo
Alopias pelagicus	Taiwan	FJ518972	2016	FJ518972_Apela_TW
Isurus oxyrinchus	Indonesia, Bali	KF793722	2015	KF793722_Ioxyr_Indo
Isurus oxyrinchus	Australia, New south wales	EU398897	2016	EU398897_Ioxyr_Aus
Atelomycterus marmoratus	Indonesia	EU398571	2016	EU398571_Amar_Indo
Cephaloscyllium umbratile	South China Sea	KP267627	2015	KP267627_Cumbr_SCS
Cephaloscyllium speccum	Australia, western	EU398675	2016	EU398675_Cspec_Aus
Cephaloscyllium variegatum	Australia, New south wales	EU398672	2012	EU398672_Cvari_Aus
Apristurus platyrhynchus	Australia, New south wales	EU398531	2012	EU398531_Aplat_Aus
Apristurus platyrhynchus	Australia, New south wales	EU398532	2012	EU398532_Aplat_Aus
Halaelurus buergeri	South China Sea	KP267642	2015	KP267642_Hbuer_SCS
Halaelurus buergeri	Malaysia	MG644339	2018	MG644339_Hbuer_My
Eridacnis radcliffei	India, Kochi	KF899421	2014	KF899421_Eradc_Ind
Eridacnis radcliffei	India, Kochi	KF899422	2014	KF899422_Eradc_Ind
Mustelus manazo	-	AB015962	2016	AB015962_Mmana
Mustelus mosis	Madagascar, Navana	HQ171693	2012	HQ171693_Mmosi_Mad

Chaenogaleus macrostoma	Kuwait	JN989312	2016	JN989312_Cmacr_Kuw
Chaenogaleus macrostoma	Iran	HQ149823	2016	HQ149823_Cmacr_Ira
Hemigaleus microstoma	Malaysia	MG644282	2018	MG644282_Hmicr_My
Hemigaleus microstoma	Indonesia, Nusa tenggara barat	EU398821	2016	EU398821_Hmicr_My
Hemipristis elongata	Indonesia, west kalimantan	KF793766	2015	KF793766_Helon_Indo
Hemipristis elongata	Indonesia, Ambon	KF590520	2015	KF590520_Helon_Indo
Carcharhinus albimarginatus	-	MT093206	2020	MT093206_Calbi
Carcharhinus albimarginatus	-	MT104516	2020	MT104516_Calbi
Carcharhinus amblyrhynchoides	Indonesia	JN034898	2016	JN034898_Camb_Indo
Carcharhinus amblyrhynchos	Australia, western	EU398598	2012	EU398598_Cambos_Aus
Carcharhinus amblyrhynchos	Indonesia, Nusa tenggara barat	EU398596	2016	EU398596_Cambos_Indo
Carcarhinus amboinensis	Indonesia, Aceh	KF590340	2015	KF590340_Cambo_Indo
Carcarhinus amboinensis	Malaysia	MG644325	2018	MG644325_Cambo_My
Carcharhinus brevipinna	Indonesia, Bali	EU398603	2016	EU398603_Cbrev_Indo
Carcharhinus dussumieri	Australia, Queensland	DQ108305	2012	DQ108305_Cduss_Aus
Carcharhinus dussumieri	Australia, western	EU398610	2012	EU398610_Cduss_Aus
Carcharhinus falciformis	Australia, Queensland	EU398614	2012	EU398614_Cfalc_Aus
Carcharhinus falciformis	Indonesia, west java	EU398613	2016	EU398613_Cfalc_Indo
Carcharhinus galapagensis	Indonesia, Java	KF590416	2015	KF590416_Cgala_Indo
Carcharhinus galapagensis	Hawaii	FJ519094	2016	FJ519094_Cgala_Haw
Carcharhinus leucas	Australia	EU818710	2016	EU818710_Cleu_Aus
Carcharhinus limbatus	Malaysia	MG644311	2018	MG644311_Clim_My
Carcharhinus macloti	Australia, Queensland	EF609312	2012	EF609312_Cmacl_Aus
Carcharhinus macloti	Indonesia, west java	EU398628	2016	EU398628_Cmacl_Indo
Carcharhinus melanopterus	Malaysia	MG644346	2018	MG644346_Cmel_My
Carcharhinus plumbeus	Indonesia, Nusa tenggara barat	EU398639	2016	EU398639_Cplum_Indo
Carcharhinus plumbeus	Malaysia	MG644376	2018	MG644376_Cplum_My
Carcharhinus sealei	Indonesia	KF590378	2015	KF590378_Csea_Indo
Carcharhinus sorrah	Malaysia	MF135191	2017	MF135191_Csor_My
Carcharhinus tjutjot	India	KU577520	2016	KU577520_Ctjut_Ind
Carcharhinus tjutjot	Indonesia, Java	JN312918	2019	JN312918_Ctjut_Indo
Galeocerdo cuvier	Indonesia, Muncar	KF590290	2015	KF590290_Gcuvi_Indo
Glyphis gangeticus	Bangladesh	MH244899	2019	MH244899_Ggang_Bang
Glyphis gangeticus	Bangladesh	MH841975	2019	MH841975_Ggang_Bang
Lamiopsis tephrodes	Indonesia	KT698047	2015	KT698047_Ltep_Indo
Loxodon macrorhinus	Malaysia	MF153996	2017	MF153996_Lmac_My
Prionace glauca	Indonesia, Java	KF793751	2015	KF793751_Pglau_Indo
Prionace glauca	Indonesia, West papua	KF793771	2015	KF793771_Pglau_Indo
Rhizoprionodon acutus	Malaysia	MF154005	2017	MF154005_Racu_My
Rhizoprionodon oligolinx	Bangladesh	MH429295	2018	MH429295_Roli_Bang
Scoliodon laticaudus	India	KF899696	2014	KF899696_Slati_Ind
Scoliodon laticaudus	Malaysia: Pasir Penambang	MW313860	2022	MW313860_Slat
Scoliodon laticaudus	Malaysia: Pasir Penambang	MW313861	2022	MW313861_Slat
Scoliodon laticaudus	Malaysia: Sungai Besar	MW313862	2022	MW313862_Slat
Scoliodon laticaudus	Malaysia: Sungai Besar	MW313863	2022	MW313863_Slat
Scoliodon laticaudus	Malaysia: Sungai Besar	MW313864	2022	MW313864_Slat

Scoliodon macrorhynchos	China	MG220559	2017	MG220559_Smac_Chi
Scoliodon macrorhynchos	Malaysia: Mukah	MW313867	2022	MW313867_Smac
Scoliodon macrorhynchos	Malaysia: Mukah	MW313869	2022	MW313869_Smac
Triaenodon obesus	Indonesia	KF590361	2015	KF590361_Tobe_Indo
Eusphyra blochii	Australia, Queensland	EU398784	2012	EU398784_Ebloc_Aus
Eusphyra blochii	Northern Australia	FJ519372	2016	FJ519372_Ebloc_Aus
Sphyrna lewini	Indonesia, Aceh	KF590347	2015	KF590347_Slewi_Indo
Sphyrna mokarran	Australia, Queensland	EU399015	2012	EU399015_Smoka_Aus
Sphyrna mokarran	Australia, western	EU399017	2012	EU399017_Smoka_Aus
Anoxypristis cuspidata	Australia, northern	EU398527	2012	EU398527_Acusp_Aus
Anoxypristis cuspidata	Indonesia, Ambon	KF590517	2015	KF590517_Acusp_Indo
Pristis pristis	Australia	GU673584	2021	GU673584_Ppris_Aus
Pristis pristis	Australia	MH005928	2018	MH005928_Ppris_Aus
Pristis zijsron	Australia, northern	EU398989	2012	EU398989_Pzijs_Aus
Rhina ancylostoma	Indonesia, Bali	EU398992	2016	EU398992_Rancy_Indo
Rhina ancylostoma	Indonesia	LC505461	2019	LC505461_Rancyl_Indo
Rhynchobatus australiae	Indonesia	MW509730	2021	MW509730_Raus_Indo
Rhynchobatus laevis	-	MN988687	2020	MN988687_Rlaev
Rhynchobatus springeri	Malaysia	MG792088	2018	MG792088_Rspri_My
Glaucostegus thouin	India, Kochi	KF899441	2014	KF899441_Gthou_Ind
Glaucostegus thouin	India, Kochi	KF899440	2014	KF899440_Gthou_Ind
Glaucostegus typus	Australia, Queensland	EU398999	2012	EU398999_Gtypu_Aus
Glaucostegus typus	Australia	HQ955939	2019	HQ955939_Gtypu_Aus
Rhinobatos jimbaranensis	Indonesia	EU398994	2016	EU398994_Rjim_Indo
Rhinobatos schlegelii	China	KP267640	2015	KP267640_Rsch_Chi
Rhinobatos formosensis	Taiwan	KP267616	2015	KP267616_Rfor_TW
Narcine brevilabiata	China	FJ237830	2016	FJ237830_Nbre_Chi
Narcine brunnea	Bangladesh	MH429319	2018	MH429319_Nbrun_Bang
Narcine maculata	India	KF899598	2014	KF899598_Nmac_Ind
Narcine cf oculifera	India, Kochi	KF899601	2014	KF899601_Ncfocu_Ind
Narcine timlei	Pakistan	MN511978	2021	MN511978_Ntiml_Pak
Dipturus kwangtungensis	Korea	EU339346	2016	EU339346_Dkwan_Kor
Dipturus kwangtungensis	Korea	EU339347	2016	EU339347_Dkwan_Kor
Okamejei boesemani	Korea	HM180813	2013	HM180813_Oboes_Kor
Okamejei boesemani	China	KP267614	2015	KP267614_Oboes_Chi
Okamejei hollandi	Taiwan	LC628959	2021	LC628959_Oholl_TW
Sinobatis borneensis	China	KX014715	2016	KX014715_Sborn_Chi
Plesiobatis daviesi	Indonesia, Java	EU398978	2016	EU398978_Pdav_Indo
Bathytoshia lata/ushiei	Indonesia, bali	EU398753	2016	EU398753_Dush_Indo
Brevitrygon heterura	Malaysia: Kuala Selangor	KM072994	2015	KM072994_Bhet2a_My
Brevitrygon heterura	Malaysia: Kuala Selangor	KM072995	2015	KM072995_Bhet1_My
Fluvitrygon kittipongi	Malaysia	MG792100	2018	MG792100_Fkit_My
Fluvitrygon kittipongi	Malaysia: Sungai Perak	MZ976814	2021	MZ976814_Fkit_My
Fluvitrygon kittipongi	Malaysia: Kuala Kangsar	MZ976815	2021	MZ976815_Fkit_My
Hemitrygon akajei	Taiwan	MG220563	2017	MG220563_Hakaj_TW
Hemitrygon akajei	Japan	JF952715	2016	JF952715_Hakaj_Jap

Hemitrygon bennetti	Malaysia: Kuala Selangor	KM073017	2015	KM073017_Hben1_My
Hemitrygon fluviorum	Australia, Queensland	DQ108183	2012	DQ108183_Hfluv_Aus
Hemitrygon fluviorum	Australia, New south wales	HM902583	2019	HM902583_Hfluv_Aus
Hemitrygon parvonigra	Indonesia, Bali	EU398732	2016	EU398732_Hpar_Indo
Himantura fava	Malaysia, Sandakan	DQ108167	2016	DQ108167_Hfava_My
Himantura leoparda	Malaysia: Sandakan	KM072996	2015	KM072996_HLEO2_My
Himantura leoparda	Malaysia: Sandakan	KM072997	2015	KM072997_HLEO1_My
Himantura uarnak	Malaysia: Semporna	KM072999	2015	KM072999_HUAR3_My
Himantura uarnak	Malaysia: Sandakan	KM073000	2015	KM073000_HUAR1_My
Himantura undulata	Malaysia	MG792127	2018	MG792127_Hund_My
Himantura undulata	Malaysia: Tawau	KM073001	2015	KM073001_HUND1_My
Maculabatis gerrardi/macrura	Bangladesh	MZ363903	2021	MZ363903_Mger_Bangladesh
Maculabatis gerrardi/macrura	Myanmar	MH235645	2018	MH235645_Mger_Myanmar
Maculabatis gerrardi/macrura	Malaysia, Kuantan	MG774924	2019	MG774924_Mmac_Kuantan
Maculabatis gerrardi/macrura	Indonesia	EU398840	2016	EU398840_Mger_IndoMuara
Maculabatis gerrardi/macrura	Malaysia, Kuantan	MG792063	2018	MG792063_Mmac_Kuantan
Maculabatis gerrardi/macrura	Malaysia, Sandakan	DQ108177	2016	DQ108177_Mger_Sandakan
Maculabatis gerrardi/macrura	Taiwan	JX263424	2016	JX263424_Mger_Taiwan
Maculabatis gerrardi/macrura	Malaysia, Sandakan	KM073002	2015	KM073002_Mmac1_MySan
Maculabatis gerrardi/macrura	Malaysia, Tawau	KM073003	2015	KM073003_Mger3_MyTaw
Maculabatis pastinacoides	Malaysia: Kuala Selangor	KM073004	2015	KM073004_MPAS1_My
Maculabatis pastinacoides	Malaysia: Sandakan	KM073005	2015	KM073005_MPAS3_My
Maculabatis toshi	Australia, Queensland	EU398864	2016	EU398864_Mtosh_Aus
Maculabatis toshi	Australia, Queensland	EU398869	2012	EU398869_Mtosh_Aus
Megatrygon microps	India, Kochi	KJ749660	2014	KJ749660_Mmicr_Ind
Megatrygon microps	India, Kochi	KJ749659	2014	KJ749659_Mmicr_Ind
Neotrygon leylandi	Australia, western	EU398751	2012	EU398751_Nleyl_Aus
Neotrygon leylandi	Australia, western	GU673442	2021	GU673442_Nleyl_Aus
Neotrygon picta	Malaysia, Pahang	MG792070	2018	MG792070_Npict_My
Neotrygon picta	Australia, Queensland	KC250625	2013	KC250625_Npict_Aus
Neotrygon malaccensis	Malacca Strait	KU497947	2016	KU497947_Nmala_MyWP
Neotrygon orientalis	Malaysia: Sandakan	KM073024	2015	KM073024_Nori2_MySan
Neotrygon orientalis	Malaysia: Sandakan	KM073025	2015	KM073025_Nori3_MySan
Neotrygon varidens	Sarawak, Malaysia	KC249902	2013	KC249902_Nvar_MySar
Neotrygon varidens	Malaysia: Kuala Selangor	KM073023	2015	KM073023_Nvar1_MySel
Pastinachus ater	Malaysia: Sandakan	KM072988	2015	KM072988_Pate1_MySan
Pastinachus ater	Malaysia, KK	EU398972	2016	EU398972_Pate_MyKK
Pastinachus gracilicaudus	Malaysia: Sandakan	KM072990	2015	KM072990_PGRA1_MySan
Pastinachus gracilicaudus	Malaysia, KK	EU398969	2016	EU398969_Pgra_MyKK
Pastinachus solocirostris	Malaysia, Mukah	EU398974	2016	EU398974_Psol_My
Pateobatis fai	Indonesia	EU398839	2016	EU398839_Pfai_Indo
Pateobatis fai	Malaysia: Semporna	KM073010	2015	KM073010_PFAI2_MySan
Pateobatis jenkinsii	Malaysia: Sandakan	KM072992	2015	KM072992_PJEN2_MySem
Pateobatis jenkinsii	Malaysia	DQ108168	2016	DQ108168_Pjenk_MySan
Pateobatis uarnacoides	Malaysia: Sandakan	KM073008	2015	KM073008_PUAC1_MySan
Pateobatis uarnacoides	Malaysia, Mukah	MG792075	2018	MG792075_Puac_MyMuk
Pteroplatytrygon violacea	Indonesia, east java	GU674230	2021	GU674230_Pviol_Indo

Pteroplatytrygon violacea	India, Kochi	KF899658	2014	KF899658_Pviol_Ind
Taeniura lymma	Malaysia: Sandakan	KM073026	2015	KM073026_TLYM2_MySan
Taeniura lymma	Malaysia, Pahang	MG792060	2018	MG792060_Tlymm_MyPah
Taeniurops meyeni	Malaysia: Sandakan	KM073019	2015	KM073019_TMEY1_MySan
Telatrygon biasa	Malaysia: Kuala Selangor	KM073020	2015	KM073020_Tbia1_MySel
Telatrygon biasa	Malaysia, Terengganu	MG774905	2019	MG774905_Tbiasa_MyTer
Urogymnus asperrimus	Australia, western	KC250636	2013	KC250636_Uaspe_Aus
Urogymnus asperrimus	India, Kochi	KT766193	2016	KT766193_Uaspe_Ind
Urogymnus granulatus	India, Kochi	KF899472	2014	KF899472_Ugran_Ind
Urogymnus granulatus	India, Kochi	KF899471	2014	KF899471_Ugran_Ind
Urogymnus polylepis	Thailand	MH908747	2019	MH908747_Upoly_Thai
Urogymnus polylepis	Malaysia: Mukah	MZ976812	2021	MZ976812_Upoly_Mk
Urogymnus polylepis	Malaysia: Sandakan	MZ976813	2021	MZ976813_Upoly_Sb
Gymnura japonica	Japan	EU398802	2016	EU398802_Gjapo_Jap
Gymnura japonica	Japan	EU398801	2016	EU398801_Gjapo_Jap
Gymnura micrura	Mexico	GU225294	2016	GU225294_Gmicr_Mex
Gymnura micrura	Mexico	HQ575767	2019	HQ575767_Gmicr_Mex
Gymnura poecilura	Malaysia, Mukah	MG792071	2018	MG792071_Gpoe_MyMuk
Gymnura zonura	Malaysia: Sandakan	KM073030	2015	KM073030_GZON1_MySan
Gymnura zonura	Malaysia: Tawau	KM073031	2015	KM073031_GZON2_MyTaw
Aetobatus flagellum	Kuwait	GU673415	2021	GU673415_Aflag_Kuw
Aetobatus flagellum	Kuwait	GU673404	2021	GU673404_Aflag_Kuw
Aetobatus ocellatus	Malaysia, Terengganu	MG774904	2019	MG774904_Aocel_MyTer
Aetobatus ocellatus	Malaysia: Sandakan	KM073028	2015	KM073028_AOCE1_MySan
Aetomylaeus maculatus	India, Kochi	KF899588	2014	KF899588_Amacu_Ind
Aetomylaeus maculatus	Malaysia, Sandakan	DQ108132	2016	DQ108132_Amacu_MySan
Aetomylaeus milvus	Qatar	GU673582	2021	GU673582_Amilv_Qat
Aetomylaeus milvus	Qatar	GU673581	2021	GU673581_Amilv_Qat
Aetomylaeus cf milvus	Kuwait	KP851151	2016	KP851151_Acmilv_Kuw
Aetomylaeus nichofii	Indonesia, kalimantan	KP851154	2016	KP851154_Anich_Indo
Aetomylaeus nichofii	Indonesia, kalimantan	KP851155	2016	KP851155_Anich_Indo
Aetomylaeus vespertilio	Australia, Queensland	EU398512	2012	EU398512_Avesp_Aus
Aetomylaeus vespertilio	India, Kochi	KF899586	2014	KF899586_Avesp_Ind
Myliobatis tobijei	South China Sea	KP267630	2015	KP267630_Mtobi_SCS
Myliobatis tobijei	Indonesia, Bali	EU398924	2016	EU398924_Mtobi_Indo
Rhinoptera javanica	Australia, Queensland	DQ108133	2012	DQ108133_Rjava_Aus
Rhinoptera javanica	Malaysia, Pahang	MG792067	2018	MG792067_Rjava_MyPah
Rhinoptera jayakari	Malaysia: Sandakan	KM073015	2015	KM073015_RJAY2_MySan
Manta birostris	Indonesia, Nusa tenggara barat	EU398904	2016	EU398904_Mbiro_Indo
Manta birostris	Indonesia, Nusa tenggara barat	GU673825	2021	GU673825_Mbiro_Indo
Mobular mobular	India, Kochi	OL714393	2021	OL714393_Mmobu_Ind
Mobular mobular	Spain	HQ956137	2016	HQ956137_Mmobu_Spa
Mobula kuhlii	India, Kochi	KF899581	2014	KF899581_Mkuhl_Ind
Mobula kuhlii	Malaysia: Sandakan	KM073011	2015	KM073011_MKUH1_MySan
Mobula thurstoni	Malaysia: Semporna	KM073012	2015	KM073012_MTHU1_MySem
Chimaera phantasma	South China Sea	KP266761	2015	KP266761_Cpha_SCS
Chimaera phantasma	Taiwan	KU687933	2017	KU68793_Cpha_TW

Urolophus expansus	Australia: Western Australia	EU399106	2012	EU399106_Uexp_Aus

Supplementary Materials Table S3. Data for the estimates of evolutionary divergence between sequences.

	N	Mean	Min	Max	SD
Intraspecific	557	0.3066	0.0000	3.8000	0.5875
Interspecific	1997	6.6003	0.6000	20.0000	3.3580
Inter-genus	4245	10.9020	3.8000	17.8000	2.6946
Inter-subfamily	4025	17.2092	13.8000	21.3000	1.2971
Inter-family	12369	16.3930	7.6000	21.9000	3.1437
Inter-order	17889	19.6294	13.2000	25.0000	1.7406
Inter-infraclass	40734	21.2381	16.9000	26.2000	1.2521
Inter-class	1620	22.3622	18.4000	25.4000	1.1050
